# Temporal profiling of the breast tumour microenvironment reveals collagen XII as a driver of metastasis

**DOI:** 10.1038/s41467-022-32255-7

**Published:** 2022-08-06

**Authors:** Michael Papanicolaou, Amelia L. Parker, Michelle Yam, Elysse C. Filipe, Sunny Z. Wu, Jessica L. Chitty, Kaitlin Wyllie, Emmi Tran, Ellie Mok, Audrey Nadalini, Joanna N. Skhinas, Morghan C. Lucas, David Herrmann, Max Nobis, Brooke A. Pereira, Andrew M. K. Law, Lesley Castillo, Kendelle J. Murphy, Anaiis Zaratzian, Jordan F. Hastings, David R. Croucher, Elgene Lim, Brian G. Oliver, Fatima Valdes Mora, Benjamin L. Parker, David Gallego-Ortega, Alexander Swarbrick, Sandra O’Toole, Paul Timpson, Thomas R. Cox

**Affiliations:** 1 The Garvan Institute of Medical Research and The Kinghorn Cancer Centre, Sydney, NSW Australia; 2 School of Life Sciences, Faculty of Science, University of Technology Sydney, Sydney, NSW Australia; 3 Cancer Ecosystems Program, Garvan Institute of Medical Research, Darlinghurst, NSW Australia; 4 School of Clinical Medicine, St Vincent’s Healthcare Clinical Campus, Faculty of Medicine and Health, UNSW Sydney, Sydney, NSW Australia; 5 Woolcock Institute of Medical Research, Respiratory Cellular and Molecular Biology, The University of Sydney, Sydney, NSW Australia; 6 Cancer Epigenetic Biology and Therapeutics, Personalised Medicine, Children’s Cancer Institute, Sydney, NSW 2031 Australia; 7 School of Women’s and Children’s Health, Faculty of Medicine, UNSW Sydney, Sydney, NSW Australia; 8 Metabolic Systems Biology Laboratory, Charles Perkins Centre, School of Life and Environmental Sciences, University of Sydney, Sydney, NSW Australia; 9 School of Biomedical Engineering, Faculty of Engineering and Information Technology, University of Technology Sydney, Sydney, NSW Australia; 10 Department of Tissue Pathology and Diagnostic Oncology, Royal Prince Alfred Hospital and NSW Health Pathology, Sydney, NSW Australia

**Keywords:** Breast cancer, Cancer microenvironment

## Abstract

The tumour stroma, and in particular the extracellular matrix (ECM), is a salient feature of solid tumours that plays a crucial role in shaping their progression. Many desmoplastic tumours including breast cancer involve the significant accumulation of type I collagen. However, recently it has become clear that the precise distribution and organisation of matrix molecules such as collagen I is equally as important in the tumour as their abundance. Cancer-associated fibroblasts (CAFs) coexist within breast cancer tissues and play both pro- and anti-tumourigenic roles through remodelling the ECM. Here, using temporal proteomic profiling of decellularized tumours, we interrogate the evolving matrisome during breast cancer progression. We identify 4 key matrisomal clusters, and pinpoint collagen type XII as a critical component that regulates collagen type I organisation. Through combining our proteomics with single-cell transcriptomics, and genetic manipulation models, we show how CAF-secreted collagen XII alters collagen I organisation to create a pro-invasive microenvironment supporting metastatic dissemination. Finally, we show in patient cohorts that collagen XII may represent an indicator of breast cancer patients at high risk of metastatic relapse.

## Abstract (web summary)

The distribution and organisation of matrix molecules in the tumour stroma help shape solid tumour progression. Here they perform temporal proteomic profiling of the matrisome during breast cancer progression and show that collagen XII secreted from CAFs provides a pro-invasive microenvironment.

## Introduction

The progression of all solid tumours is a function of both intrinsic cellular mutational burden and extrinsic effectors provided by the tumour microenvironment^1^. Analogous to organ development, breast cancer progression depends on an interplay between the proliferating breast cancer cells and the biochemical and biophysical properties of the extracellular matrix (ECM)^2^. The ECM is a dynamic 3D supramolecular network, essential for coordinating multicellular life. The continually changing ECM during cancer progression shapes and defines the tumour microenvironment, altering both tumour growth and metastatic dissemination.

In cancer, the normal tissue matrix is progressively replaced by the tumour matrix, which is primarily secreted by cancer-associated fibroblasts (CAFs)^3–7^. Of particular note is collagen I, a major structural component of the ECM that has been extensively studied and its abundance has been associated with both pro-tumourigenic^8–13^, as well as anti-tumourigenic roles^14,15^ in different solid tumour types. Furthermore, increased collagen I deposition significantly contributes to high mammographic density^16–19^, a known risk factor for aggressive breast cancer, correlating with poor clinical outcomes^20–23^. Previous studies on the influence of collagen I abundance in accelerating or suppressing tumour progression point to a more nuanced understanding of the matrix in regulating tumour progression. In particular, the association of collagen I architecture with patient outcome in breast cancer^12,13,24^, suggests that collagen I architecture and not merely abundance is a critically important regulator of tumour progression.

Collagens are typically secreted and assembled extracellularly during the genesis and repair of the 3D matrix. The deposition and organisation of collagen I fibres and fibrils are regulated by other matrix components, such as through interactions with the proteoglycans decorin^25^, tenascin X^26^, the glycoprotein fibronectin^27^, as well as the family of fibril-associated collagens with interrupted triple helices (FACIT) collagens^28–30^. Capturing the diversity and complexity of this ECM network requires matrix-specific sample preparation and analytical methods^31–35^, and has provided fundamental insights into matrix characteristics of the stem cell niche^32^, tumours^33^ and their metastatic sites^4^, for example. Temporal profiling has the potential to reveal dynamic re-organisation of the matrix throughout development and disease progression.

In the context of cancer, the non-selective depletion of the matrix^36–38^, and/or matrix-producing cells^15^, has been shown to have adverse outcomes and can, paradoxically, accelerate tumour progression and metastatic dissemination. An emerging understanding of how the matrix network changes during tumour progression will provide insight into the co-operativity of matrix molecules to regulate cancer cell behaviour. Furthermore, with this in mind, more nuanced approaches focussed on matrix normalisation or re-engineering to restore the composition and architecture of healthy tissues are therefore likely to offer greater promise in the therapeutic setting. Indeed, previous work has shown that collagen cross-linking by lysyl oxidases and changes in tumour stiffness is critically important in driving the progression of breast cancer and that blocking this cross-linking can block tumour progression^9^. Further work has also shown that the biophysical properties of the ECM can also modify treatment response in triple-negative breast cancer^39^, and agents that modulate stiffness-dependent NF-kB or JNK activity could enhance therapeutic efficacy in TNBC patients^40^ as well as in other cancer types^41^. Therefore, uncovering elements which regulate matrix assembly and organisation, and the role that they play in solid tumour progression will be critical to dissecting the role of the matrix in cancer^1^.

However, more work is needed to understand how matrix composition, including the amount of matrix, and in particular the source of secretion (cancer cells versus stromal cells^3,42^) contribute to the observed pro- and anti-tumourigenic effects on cancer development and progression. It is likely that the balance between pro- and anti-tumourigenic matrix cues likely tips in favour of tumour- and metastasis-promoting roles as solid tumours progress, and as such understanding the timing of these events will also be critical in designing future interventions.

In this work, through coupling of our ISDoT tissue decellularisation technology with quantitative mass spectrometry^31^, we profile the changing tumour matrisome during mammary tumourigenesis in the PyMT breast cancer model, identifying temporal changes in the breast cancer matrisome. Through our analysis, we identify collagen XII, a FACIT matrix protein important in regulating collagen I assembly and organisation in load-bearing tissues such as skeletal muscle and tendon as well as the cornea^30,43–46^, as heavily implicated in mammary tumour progression. Collagen XII has been identified as a marker of poor prognosis in colorectal cancer^47,48^, although the underlying mechanism is unclear and to date its functional role in breast cancer remains underexplored. We demonstrate that collagen XII is predominantly secreted by CAFs and regulates collagen I fibril organisation to promote cancer cell invasion and breast cancer metastasis. This functional characterisation of collagen XII’s importance in breast cancer metastasis is in addition to proteomic data detailing temporal changes in the ECM of breast tumours throughout progression and together warrants further investigation into collagen XII as a potential marker for breast cancer patients at high risk of relapse.

## Results

### Temporal proteomic profiling of the tumour matrisome

**Fig. 1:**
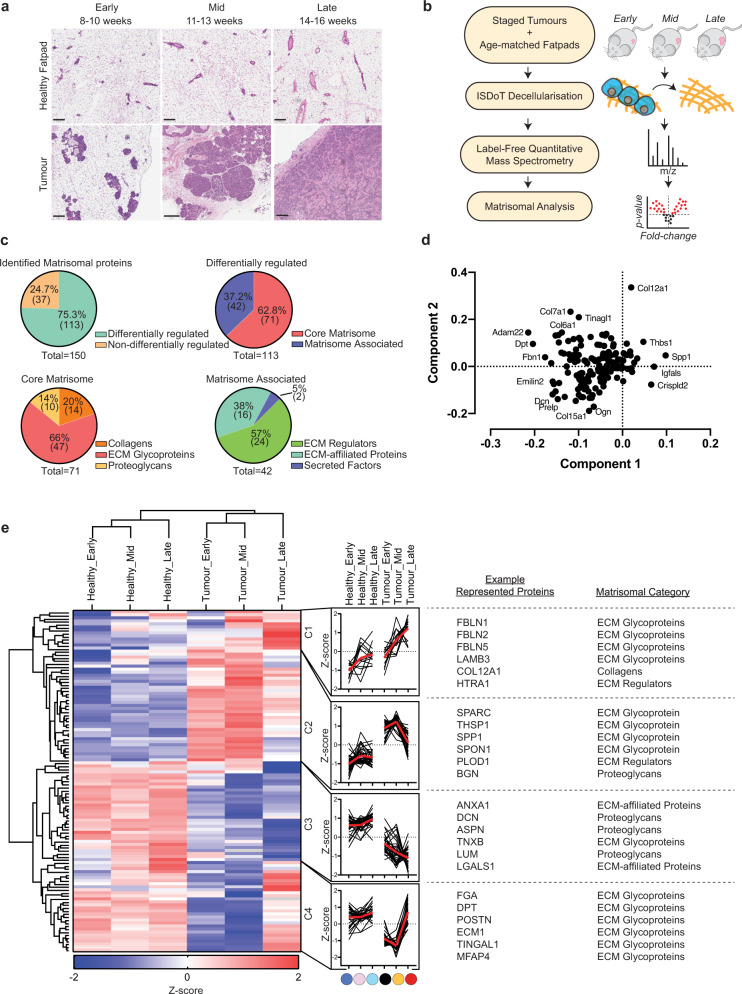
Proteomic profiling of decellularized breast tumours reveals dynamic changes in the matrisome. **a** Histological images (H&E) of mammary tumours and age-matched healthy fatpads from early (8–10 weeks), mid (11–13 weeks) and late (14–16 weeks) stages (scale bar = 200 µm). Representative images of *n* = 4 mice for the tumour group at mid stage and *n* = 5 mice for all other groups. **b** Decell label-free quantitative liquid chromatography-tandem proteomic mass spectrometry (LC-MS/MS) workflow. **c** Breakdown of matrisome classes^35^ detected in tumours and matched healthy fatpads identified by LC-MS/MS. **d** Principal component analysis (PCA) biplot of tumour and matched healthy fatpad matrisome data. **e** Temporal profiling of mammary tumours and matched healthy fatpad matrisome. (left) Two-dimensional unsupervised hierarchical clustering of tumours and matched healthy fatpad by Euclidean distance demonstrating 4 clusters (C1–C4) with corresponding profile plot beside each cluster (log_2_ transformed, median-centred *z*-score of protein abundance values from proteins present in at least 70% of all samples, median of replicate samples from each condition *n* = 4–5). (right) Representative example proteins and corresponding matrisome subcategories. Differentially abundant proteins in LC-MS/MS were determined by multi-sample ANOVA (FDR = 0.05). All data derived from *n* = 4 mice for the tumour group at mid stage and *n* = 5 mice for all other groups. Source data are provided in the Source data file.

As solid tumours grow, the ongoing remodelling of the ECM leads to the loss of ‘normal’ tissue matrix and replacement with tumour matrix. This remodelling plays a key role in tumour progression^1^. We profiled the temporal changes in ECM of developing breast tumours in the spontaneous polyoma middle-T (PyMT) genetically engineered mouse model (GEMM), from early- (hyperplasia), mid- (adenoma), and late- (metastatic adenocarcinoma) stages, along with age-matched mammary fatpads from healthy mice. Tissues were collected at early (8–10), mid (11–13), or late (14–16 weeks) stages of development (Fig. 1a). As an immunocompetent breast cancer model that mimics the molecular and histological progression of human breast cancer through premalignant stages and metastatic dissemination, this model can provide key insight into tumour biology throughout breast cancer progression including a fibroblastic stroma. Gene expression profiling has revealed that the PyMT model recapitulates many aspects of hormone receptor-negative human breast cancers that have the poorest prognosis^49^. Tissues were decellularized in line with our previous work^31^ to enrich ECM proteins before label-free quantitative liquid chromatography-tandem mass spectrometry (LC-MS/MS) (Fig. 1b) was undertaken. A total of 264 matrisomal components were consistently detected across all sample datasets (workflow shown in Supplementary Fig. 1A). Of these, 150 were robustly quantified in matched healthy fatpad and tumour samples (Fig. 1c top left, Supplementary Data 1). 113 matrisomal elements were determined to be differentially abundant by multi-sample ANOVA (false discovery rate = 0.05), either temporally (early–mid–late), and/or between healthy fatpad and tumour at any given stage of development. Approximately 63% (71/113) were categorised as core matrisome proteins (Fig. 1c top right), comprising proteoglycans, collagens, and glycoproteins (Fig. 1c bottom left). The remaining molecules were matrisome-associated, including secreted factors, ECM-affiliated proteins, and ECM regulators (Fig. 1c bottom right).

Principal component analysis of samples and visualisation of the loadings for Principal components 1 (PC1) and 2 (PC2) identified a number of proteins that most strongly contributed to clustering (Fig. 1d and Supplementary Fig. 1B). Unsupervised hierarchical clustering of log_2_ transformed, median-centred, *z*-scored data revealed four discrete clusters with distinct temporal profiles (C1–C4) (Fig. 1e left). C1 represents proteins which are progressively upregulated over time in healthy tissues, yet are significantly over-expressed in tumours and increase further with tumour progression. C2 demonstrates proteins which are consistently upregulated at all stages of tumour development compared to healthy fatpad. C3 includes proteins which are progressively downregulated during disease progression, and C4 represents proteins downregulated in early and mid-stages of development but increase significantly in the later stage compared to normal fatpad. Fisher’s exact testing showed no significant association of matrisome categories within each temporal clusters.

Confirming previously published work, we note that the matrix glycoprotein osteopontin (SPP1) is highly abundant at the primary breast tumour site (Fig. 1e right; C2), a matrix element which has been shown to activate mammary fibroblasts into pro-tumourigenic cancer-associated fibroblasts (CAFs)^50^. Furthermore, the proteoglycan decorin (DCN), which is known to be downregulated in breast cancer and has demonstrated both anti-proliferative and anti-metastatic properties^31,51,52^, was significantly reduced in late-stage tumours compared to healthy fatpad in our data (Fig. 1e right; C3). We also observed the downregulation of tubulointerstitial nephritis antigen-like 1 (TINAGL1) (Fig. 1e right; C4), which has been previously shown to exhibit a tumour suppressive role in breast cancer through binding to epithelial integrin α5β1, αvβ1, and epidermal growth factor receptor (EGFR), inhibiting focal adhesion kinase (FAK) and EGFR signal transduction^53^. Finally, we observed downregulation of tenascin X (TNXB) at all stages of tumour progression (Fig. 1e right; C3), which has recently been reported to be downregulated across numerous solid cancer types and associates with poor survival^54^ and may represent a pan-cancer marker. Together these data validate our approach and highlight how key ECM molecules may play important roles in shaping the progression of primary breast tumours, from fostering tumour cell proliferation and invasion into the stroma, migration toward the vasculature, intravasation and vascular dissemination leading to metastatic disease^1,2^.

### Collagen XII shows temporal upregulation during mammary carcinoma development

**Fig. 2:**
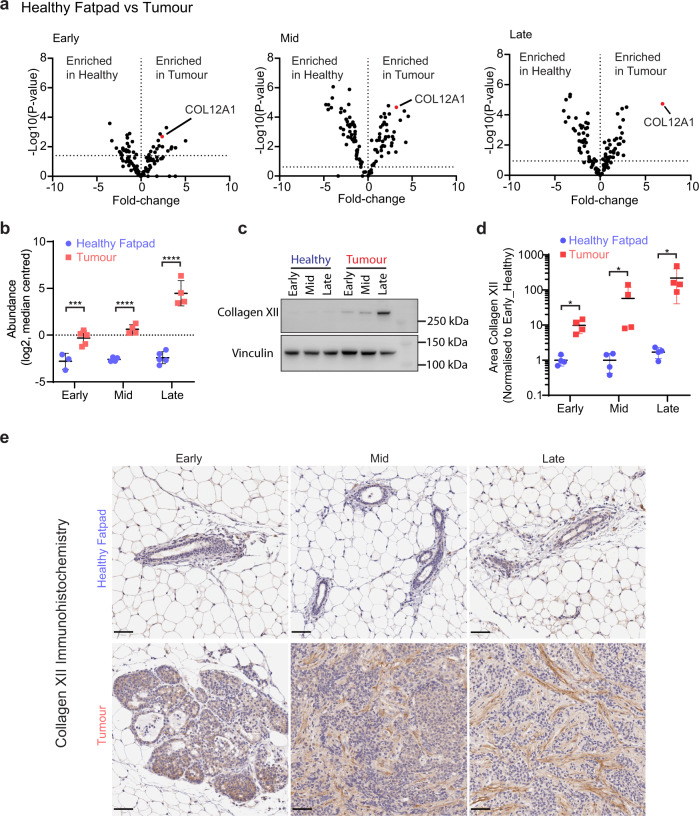
Collagen XII is upregulated in breast tumours as the disease progresses. **a** Volcano plot of differentially abundant matrisome proteins from LC-MS/MS in matched healthy fatpad or mammary tumour at the early, mid or late stages highlighting collagen XII elevation in mammary tumours at each stage. Multi-sample *t* test with FDR correction. Data derived from *n* = 4 mice for the tumour group at mid stage and *n* = 5 mice for all other groups. **b** Collagen XII protein abundance in tumour and matched healthy fatpad tissue, quantified by LC-MS/MS (log_2_ transformed, median centred, collagen XII was detected in *n* = 25 of 30 samples corresponding to tissue samples from *n* = 3 mice in the healthy early stage group, *n* = 4 mice in the healthy mid, tumour mid and tumour late stage groups and *n* = 5 mice from the healthy late and tumour early stage groups). ***p = 0.0003, ****p < 0.0001 One-way ANOVA with Holm–Sidak multiple comparison test. Data are presented as mean ± SD. **c** Temporal profile of collagen XII protein expression in healthy fatpad and primary tumours via western blot. Representative of *n* = 3 biologically independent samples. **d** Quantification of collagen XII immunohistochemistry (IHC) staining of staged tumours and matched healthy fatpad tissue. Average of 5× fields of view per tumour in *n* = 4 mice per group. *p = 0.029 Mann–Whitney *U*-test (two-sided). Data are presented as mean ± SD. **e** Representative histological images of collagen XII staining quantified in **d**). Scale bar = 50 µm from *n* = 4 biologically independent samples. Source data are provided in the Source data file.

The treatment of patients with localised primary breast tumours has significantly improved patient outcome in recent years, yet predicting metastatic risk and the treatment of these patients remain a significant clinical problem. When comparing expression changes of matrisome components in healthy fatpad to tumour matrisomes at each stage, we found a number of differentially abundant ECM proteins (Fig. 2a). We were interested in those that were not only robustly detected across all samples, but also those that were lowly and stably expressed in healthy fatpad tissue, and increasingly upregulated as tumours progressed, specifically those in cluster 1 (C1) (Fig. 1e). Collagen XII (COL12A1) demonstrated highly significant up-regulation in tumours at all stages of development compared to healthy controls (Fig. 2a). Furthermore, COL12A1 was identified in our PCA loading analysis as the strongest ECM component contributing to the second principal component (PC2) (Fig. 1d), which separated late- from early-stage tumours (Supplementary Fig. 1B), suggesting it may be strongly associated with tumour progression. Elevated expression of COL12A1 in breast tumours was also supported by our previous ISDoT-based global proteomic profiling of the matrisome in the syngeneic immunocompetent 4T1-Balb/C model where there is an elevated expression of collagen XII in tumours compared to healthy age-matched control^31^. Furthermore, visualisation of the temporal expression profile from early- to mid- to late-stage disease revealed that collagen XII abundance was progressively upregulated as primary tumours developed (Fig. 2b), which was validated by western blot of tissue lysates (Fig. 2c and Supplementary Fig. 1C, D). The spatial expression pattern of collagen XII in primary tumours was evaluated by immunohistochemistry (IHC) and exhibited a predominantly stromal localisation which increased with tumour stage (Fig. 2d, e), confirming its role as a key matrix scaffolding protein^30,45,46^.

Whilst collagen XII is a known regulator of fibrillar collagen architecture in normal tissues^30^ and has been correlated with tumour aggressiveness^12^, a direct role for this matrix component in regulating breast tumour progression remains to be defined.

### Collagen XII expression associates with changing tumour biomechanics

Collagen XII is the largest member of the fibril-associated collagen with interrupted triple helices (FACIT) collagens^30^. Collagen XII is known to bind to type I collagen fibrils and regulate their organisation and consequently contribute to tissue biomechanics in the cornea, skeletal muscle and tendon tissues^30,43–46^. Recently, it was elegantly shown that collagen XII expression is associated with high mammographic density in women (a known risk factor of aggressive breast cancer), which is itself associated with increasing matrix stiffness and collagen I bundle thickness^20^. Collagen XII has also previously been associated with clinical outcomes in gastric cancer^55^. To evaluate the potential role of collagen XII in primary tumour progression, we characterised collagen I/collagen XII spatial organisation over time, as well as tumour biomechanics.

**Fig. 3:**
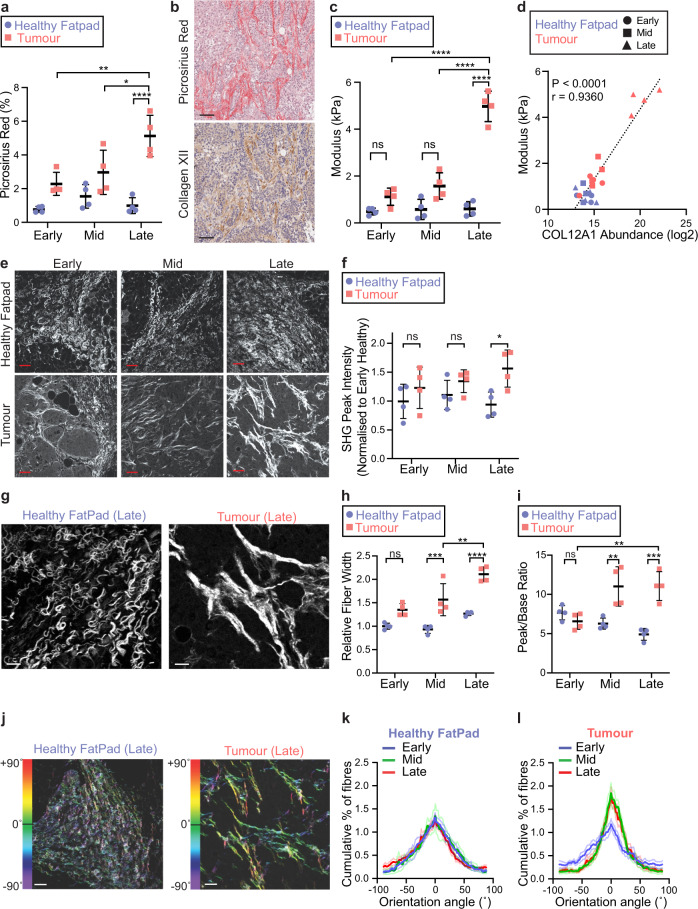
Collagen XII abundance is associated with altered fibrillar collagen architecture and tumour stiffness. **a** Quantification of total picrosirius red stained area in tumour and healthy fatpad tissues. *n* = 4 tissues; *p = 0.023, **p = 0.0022, ****p < 0.0001; two-way ANOVA with Tukey’s multiple comparison test. Mean ± SD presented. **b** Representative images of serial histological sections from *n* = 4 mammary tumour tissues stained for fibrillar collagen (picrosirius red), and collagen XII by IHC (scale bar = 50 µm). **c** Unconfined compression analysis of *n* = 4 tumours and healthy fatpads (mean ± SD, *n* = 4. ****p < 0.0001, ns = not statistically significant; early healthy vs. early tumour *p* = 0.37; mid healthy vs. mid tumour *p* = 0.051; two-way ANOVA with Tukey’s multiple comparison test. **d** Correlation between collagen XII protein abundance (LC-MS/MS) and bulk modulus of primary tissues (Pearson correlation test, *r* = 0.936, *p* < 0.0001; *n* = 24 tissues). **e** Representative single-plane images of second harmonic generation (SHG) signal acquired from *n* = 4 tumours and healthy fatpad tissues at early, mid and late stages (scale bar = 40 µm). **f** Quantification of peak SHG multi-photon signal. *n* = 4 tissues per group; *p = 0.017, ns = not statistically significant: early healthy vs. early tumour *p* = 0.58; mid healthy vs. mid tumour *p* = 0.57. Two-way ANOVA with Tukey’s multiple comparison test. **g** Representative single-plane images of SHG multi-photon images acquired from *n* = 4 late-stage tumours and healthy fatpad tissues (scale bar = 15 µm). **h** Quantification of collagen I fibre bundle width in images from **g**. Mean ± SD of *n* = 4 tissues per group; **p = 0.0032, ***p = 0.0006, ****p < 0.0001, ns: *p* = 0.083; two-way ANOVA with Tukey’s multiple comparison test. **i** Quantification of collagen I fibril orientation peak to baseline ratio. Mean ± SD, *n* = 4 tissues per group; **p < 0.01, ***p < 0.001, ns: *p* = 0.89; mid healthy vs. mid tumour *p* = 0.0025, late healthy vs. late tumour *p* = 0.0001, early tumour vs. late tumour *p* = 0.0042; two-way ANOVA with Tukey’s multiple comparison test. **j** Representative images of fibril-orientation analysis for collagen I fibres in *n* = 4 tissues (scale bar = 40 µm). Collagen I fibril orientation distribution for **k** staged healthy fatpad and **l** tumour (±SEM, *n* = 4) corresponding to analysis in **i**. Source data are provided in the Source data file.

Picrosirius red binds to, and stains fibrillar collagens present within tissues^56,57^. Using serial picrosirius red stained and collagen XII IHC stained sections from tumours we confirmed that total collagen increased along with collagen XII during tumour progression (Fig. 3a, b and Supplementary Fig. 2A, B). The co-localisation of collagen XII with fibrillar collagen supports the known role of collagen XII in stabilising collagen I fibrils and regulating their 3D organisation in the cornea, skeletal muscle and tendon^30,43–46^. Using unconfined compression analysis to measure the biomechanical properties of tumours, we confirmed that tumour stiffness (bulk elastic modulus) increased significantly in the late stages of disease (Fig. 3c), correlating directly with the observed increase in collagen XII abundance quantified by mass spectrometry (Fig. 3d).

Exploiting the non-centrosymmetric nature of collagen I fibrils, which allows for label-free imaging and quantification using second-harmonic generation (SHG) multi-photon imaging^58,59^, we quantified the differences in the organisation of collagen I fibrils and fibres in tumours and healthy fatpad tissues (Fig. 3e). Quantification of SHG peak signal showed an upregulation of collagen I fibre density in the late stage of tumour development (Fig. 3f) matching the increase observed in picrosirius red staining. Quantification of collagen I fibre bundle dimensions revealed an increase in collagen I bundle width as tumours progressed (Fig. 3g, h). Finally, analysis of collagen I fibre orientation^31^ demonstrated an increase in collagen I fibre linearity as tumours progressed from early to mid and late stages (Fig. 3i–l). Together these data confirm a widespread remodelling of fibrillar collagen I organisation during primary tumour progression.

### Collagen XII is upregulated in human breast cancer and is associated with a poor prognosis

**Fig. 4:**
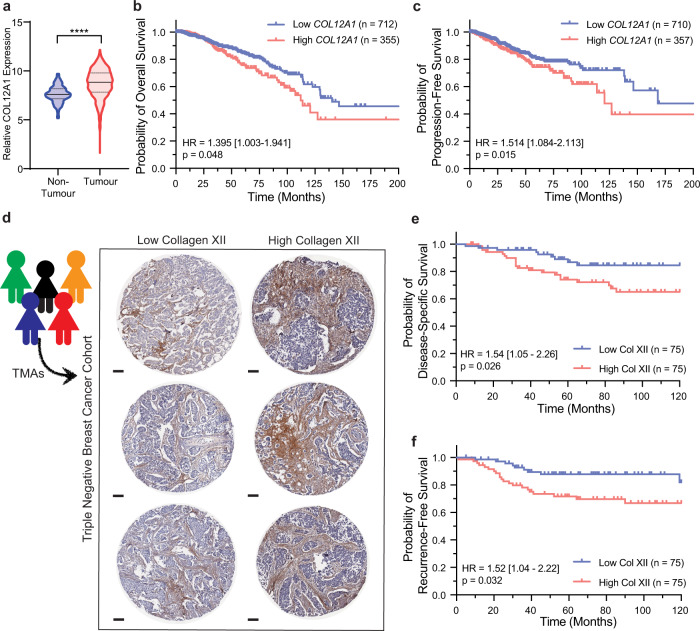
Collagen XII expression is associated with poor prognosis in human breast cancer. **a** *COL12A1* expression in human non-tumour mammary tissue (*n* = 98) or primary tumour (*n* = 1080) from ‘The Cancer Genome Atlas’ (TCGA) breast adenocarcinoma cohort (BRCA)^60^ (Median and interquartile range, *p* value determined by two-sided Mann-Whitney test (****p < 0.0001 Mann–Whitney *U*-test). **b** Kaplan–Meier overall survival and **c** progression-free survival analysis of collagen XII stratified (tertiles) breast cancer patients from TCGA BRCA dataset (*n* = 1067). Univariate Cox Proportional Hazards Model hazard ratio (HR), HR 95% confidence interval and *p* value for collagen XII high vs low are indicated. **d** Representative collagen XII IHC-stained TMA sections from triple-negative human breast cancer (TNBC) cohort^61^ of *n* = 150 patients (scale bar = 100 µm). **e** Kaplan–Meier analysis of disease-specific survival and **f** distant recurrence from TNBC TMA sections, median-stratified by % stromal collagen XII staining (*n* = 150, 3 TMA cores per patient). Univariate Cox Proportional Hazards Model hazard ratio (HR), HR 95% confidence interval and *p* value for collagen XII high vs low are indicated. Source data are provided in the Source data file.

To determine the clinical relevance of collagen XII in breast cancer patients, collagen XII expression was analysed in RNAseq data from the Cancer Genome Atlas (TCGA) breast cancer cohort (BRCA)^60^. This confirmed significant upregulation of collagen XII across all primary breast tumour types, including TNBC, relative to non-tumour tissue (Fig. 4a). Kaplan–Meier analysis of collagen XII gene expression and patient survival in the same dataset^60^ demonstrated that high collagen XII expression in the primary tumour is significantly associated with both poor overall survival (OS) (Fig. 4b and Supplementary Table 1) and poor progression-free survival (PFS) (Fig. 4c and Supplementary Table 2). Multivariate analysis of collagen XII expression together with corrections for the clinical covariates age, stage, receptor status and the presence of cancer cell positive lymph nodes revealed that collagen XII expression was the strongest predictor for progression-free survival in early stage (Stages I and II) breast cancer compared to these clinical covariates (Supplementary Table 3). However, collagen XII expression was not a significant predictor of overall or progression-free survival when tumours across all stages (I–IV) were examined together (Supplementary Tables 3 and 4), suggesting that collagen XII expression is more strongly associated with early-stage progression.

To further confirm these findings, we performed collagen XII immunohistochemistry (IHC) on a patient tissue microarray (TMA) cohort containing 150 breast cancer patients with comprehensive clinicodemographic and follow-up data^61–63^. The TMAs contained at least three cores taken from different areas of each patient tumour (Fig. 4d). Our analysis revealed that high collagen XII stromal staining (measured as the % positivity of tumour stroma) was significantly associated with a poor disease-specific-survival (Fig. 4e and Supplementary Table 5), and also with a higher incidence of distant recurrence (Fig. 4f and Supplementary Table 6). Of note is that additional Cox Proportional Hazards multivariate modelling of distant recurrence in this TMA dataset revealed that collagen XII staining is significantly associated with distant recurrence independently of significant clinical risk factors including lymphatic invasion (*p* = 0.034) (Supplementary Table 7). The strong association of collagen XII expression with patient outcome, and in particular recurrence in early-stage patients, warrants further investigation to determine its potential as a biomarker of aggressive metastatic disease.

### Single-cell transcriptomics reveal CAFs underpin collagen XII levels in the tumour microenvironment

**Fig. 5:**
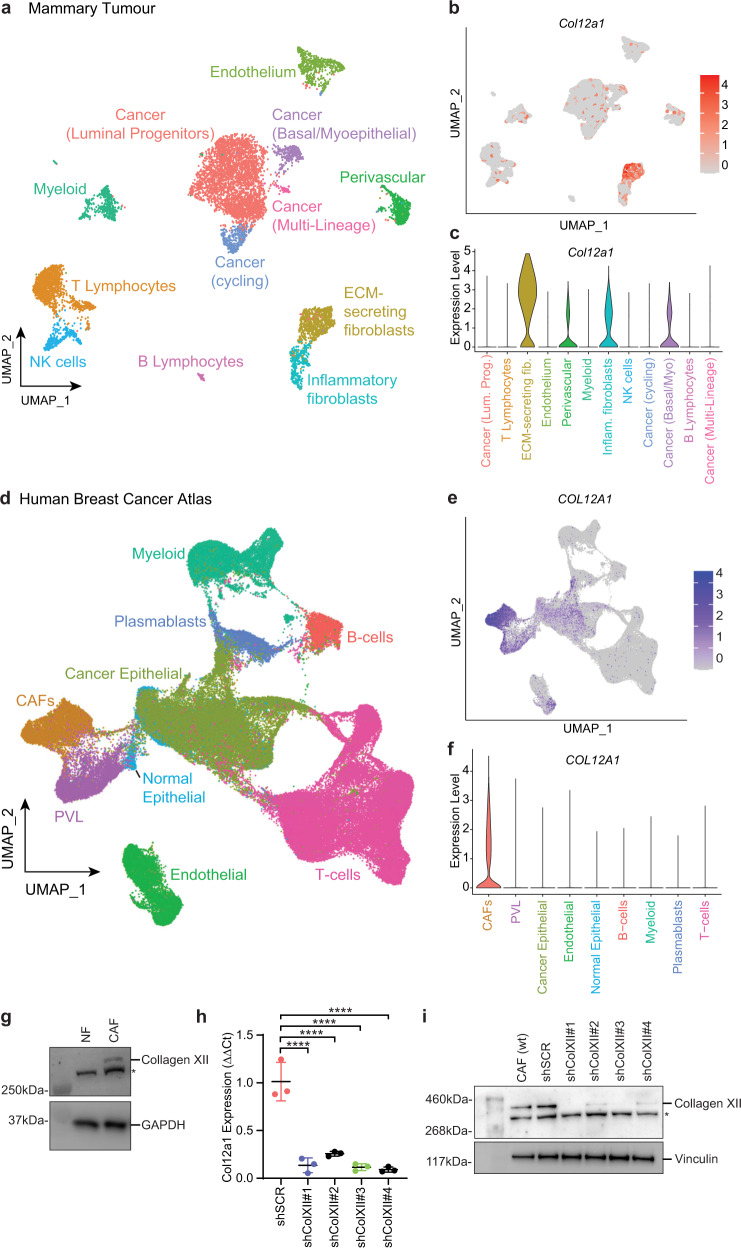
Collagen XII is produced by cancer-associated fibroblasts in mouse and human breast tumours. **a** UMAP visualisation and clustering of 11,490 cells analysed by single-cell RNA-sequencing from Valdes-Mora et al.^66^ of late-stage mammary tumours. **b** Feature plot and **c** Violin plot showing the log-normalised expression of *Col12a1* over the UMAP structure and clusters as shown in **a** confirming the source of collagen XII expression. **d** UMAP visualisation and clustering of 100,064 cells analysed by single-cell RNA-Sequencing from the Breast Cancer Atlas^67^, a study of 26 human primary breast cancers. Cell clusters are labelled by their major lineage annotations for B cells, Plasmablasts, T cells, Myeloid, Cancer epithelial, Normal epithelial, cancer-associated fibroblasts (CAFs), perivascular-like cells (PVL) and endothelial cells. **e** Feature plot and **f** Violin plot showing the log-normalised expression of *COL12A1* over the UMAP structure and major lineage clusters as shown in **d**. **g** Western blot analysis of collagen XII secretion by normal mammary fibroblast (NF) and cancer-associated fibroblasts (CAFs) (*denotes non-specific band). GAPDH is loading control. Representative of *n* = 3 biologically independent experiments. **h** Quantification of *Col12a1* mRNA expression (*n* = 3 biologically independent experiments ****p < 0.0001 one-way ANOVA with Tukey’s multiple comparison test) and **i** collagen XII protein expression (representative of *n* = 3 biologically independent experiments) in collagen XII knockdown CAF lines generated using collagen XII short-hairpin mRNA scrambled control (shScr) and four collagen XII targeting constructs (shColXII#1–#4) (*denotes non-specific band). Vinculin is loading control. Source data are provided in the Source data file.

The major architects of remodelling of the ECM in solid tumours are known to be CAFs^64,65^. To assess the source of collagen XII within the primary tumour microenvironment, we analysed single-cell RNAseq (scRNA-seq) of five primary late-stage tumours collected from our in vivo models^66^. These data confirmed that the predominant producers of collagen XII (*Col12a1*) are the matrix-secreting subtype of cancer-associated fibroblasts (CAFs) in this model (Fig. 5a–c). To further validate the relevance of this finding to the human setting, we also analysed scRNA-seq data from a cohort of 26 primary breast tumours^67^ of the three major clinical breast cancer subtypes (ER+, TNBC and HER2+) sampled from patients undergoing surgery. scRNA-seq analysis of 100,064 cells (Fig. 5d, e) further confirmed that CAFs are the major producers of collagen XII within primary human breast tumours (Fig. 5f).

The presence of CAFs within breast tumours has previously been associated with poor prognosis^64,68^. To investigate the relationship between *COL12A1* and CAF presence in human tumours we assigned a CAF score to each breast tumour dataset from the TCGA cohort using the CAF marker genes identified in human scRNA-seq data from breast tumours above (Fig. 5f). Our analysis identified a strong positive correlation between *COL12A1* expression and CAF score, further reinforcing CAFs as the major source of *COL12A1* in breast tumours (Supplementary Fig. 3A). In vitro and in vivo studies have demonstrated that CAFs can potentiate tumour progression^64,68^ and so in order to dissect the contribution of CAFs from that of collagen XII expression in the RNAseq data, we compared models of overall and progression-free survival with or without corrections for CAF score. Our analyses show that collagen XII expression, but not the CAF score, is significantly associated with overall and progression-free survival (Supplementary Table 8). Furthermore, we observe that the addition of the CAF score to the collagen XII survival model does not significantly improve the model (Supplementary Table 9), indicating that collagen XII expression is a predictor of outcome in these patients independently of the CAF score.

### CAF-secreted collagen XII modulates collagen I organisation, tissue biomechanics and cancer cell invasion

To study the functional role of collagen XII in primary breast tumour progression in our model, we used CAFs and cancer cells isolated from primary PyMT tumours^10^. We confirmed collagen XII upregulation in these CAFs relative to normal murine mammary fibroblasts (NFs) (Fig. 5g and Supplementary Fig. 4A, B) in line with scRNA-seq data. We then generated stable collagen XII knockdown CAFs using four individual *COL12A1* short-hairpin RNA (shRNA) constructs (shColXII #1–#4). Following selection, stable knockdown was validated at both the mRNA (Fig. 5h) and protein (Fig. 5i and Supplementary Fig. 4C, D) level. Two independent lines (shColXII #1 and #3) showing significant collagen XII knockdown, plus control (shSCR)(with similar expression to the parental line) were used for functional validation of CAF-secreted collagen XII in primary breast cancer development and metastasis.

**Fig. 6:**
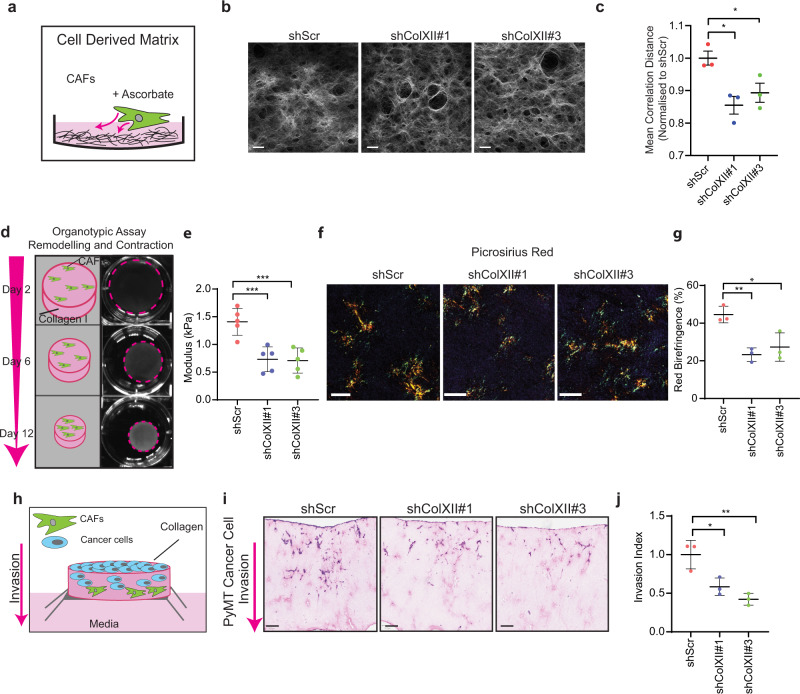
Collagen XII knockdown modulates fibrillar collagen architecture and inhibits cancer cell invasion. **a** Schematic representation of cell-derived extracellular matrix (CDM) secreted by cancer-associated fibroblasts (CAFs). **b** Representative single-plane images of second harmonic generation (SHG) multi-photon signal of CDMs produced by CAF scrambled control (shScr) and two stable collagen XII knockdown lines (shColXII#1 and #3) from *n* = 3 biologically independent experiments. **c** Grey-level co-occurrence matrix (GLCM) analysis of mean correlation distance of collagen I structure in CAF-derived CDMs following collagen XII knockdown. Mean  ± SD, *n* = 3 biologically independent experiments, *p < 0.05, shScr vs. shColXII#1 *p* = 0.14; shScr vs. shColXII#3 *p* = 0.049; One-way ANOVA with Dunnett’s multiple comparisons test. **d** Schematic representation of 3D organotypic matrix remodelling by fibroblasts (scale bar = 7 mm). Representative of *n* = 5 biologically independent experiments. **e** Bulk modulus (stiffness) of organotypic matrices remodelled by control (shScr) or collagen XII knockdown (shColXII#1, shColXII#3) CAFs at day 12 as measured by unconfined compression analysis. Mean ± SD, *n* = 5 biologically independent experiments, ***p < 0.001, shScr vs. shColXII#1 *p* = 0.0006; shScr vs. shColXII#3 *p* = 0.0008; one-way ANOVA with a Dunnett’s multiple comparisons test. **f** Representative images of birefringence signal from picrosirius-red-stained organotypic matrices generated by control (shScr) or collagen XII knockdown (shColXII#1, shColXII#3) CAFs imaged under polarising light (scale bar = 100 µm) from *n* = 3 biologically independent experiments. **g** Quantification of the red birefringence signal area for picrosirius-red-stained organotypic matrices remodelled by control (shScr) or collagen XII knockdown (shColXII#1, shColXII#3) CAFs. Mean ± SD, *n* = 3 biologically independent experiments, *p = 0.014, **p = 0.0054, one-way ANOVA with a Dunnett’s multiple comparisons test, **h** Schematic representation of experimental set-up of cancer cell invasion into CAF-remodelled organotypic matrices. **i** Representative histological images (H&E) (*n* = 3) of PyMT cancer cell invasion into organotypic matrices remodelled by control (shScr) or collagen XII knockdown (shColXII#1, shColXII#3) CAFs (scale bar = 100 µm). **j** Quantification of PyMT cancer cell invasive index into organotypic matrices. Mean ± SD, *n* = 3 biologically independent experiments, *p = 0.020, **p = 0.0042, One-way ANOVA with a Tukey’s multiple comparison test. Source data are provided in the Source data file.

First, we sought to determine the effects that collagen XII knockdown had on de novo synthesis of cell-derived matrices (CDMs)^69–71^ (Fig. 6a). Imaging of collagen I in CDMs using SHG multi-photon imaging (Fig. 6b) showed no significant changes in the total amount of collagen I deposited, as measured by SHG peak signal intensity (Supplementary Fig. 5A). However the spatial patterning of the SHG signal, as measured by grey-level co-occurrence matrix (GLCM) analysis^72–75^ of the collagen fibres, showed a significant decrease in the uniformity of the collagen network following collagen XII knockdown (Fig. 6c). Importantly, these data indicate that knockdown of collagen XII expression does not alter the total abundance of fibrillar collagen, but does change fibrillar collagen ultrastructure. These data confirm that collagen XII is playing a role in determining spatial organisation of collagen I fibrils, and that the depletion of collagen XII in CAFs may act to normalise the matrix.

The 3D organisation and interaction of collagen family members are critically important in regulating the properties of the 3D microenvironment. In particular, changes in collagen I organisation are known to influence the biomechanical properties of tissues^16^, a parameter which has been shown to be increasingly important in solid tumour biology^8^. To determine the effect of collagen XII on collagen I organisation and biomechanics in 3D, shScr, shColXII#1 and shColXII#3 CAFs were seeded into and allowed to remodel, 3D collagen I organotypic matrices^72–74,76^ (Fig. 6d). Collagen XII knockdown did not alter CAF ability to contract the organotypic matrices (Supplementary Fig. 5B, C). However, unconfined compression analysis of the bulk biomechanical properties of the organotypic matrices, revealed that collagen XII depletion in CAFs led to a decrease in overall matrix stiffness (Fig. 6e).

Picrosirius red staining and polarised light birefringence imaging of the remodelled organotypic matrices (Fig. 6f) demonstrated a significant decrease in red (Fig. 6g) and yellow (Supplementary Fig. 5D) bundles as well as a corresponding increase in thin (green) fibrillar collagen bundles (Supplementary Fig. 5E) in the shColXII#1 and shColXII#3 matrices compared with shScr matrices. These data indicate that the depletion of collagen XII leads to a higher abundance of thinner collagen bundles. As collagen XII scaffolding underpins collagen I bundling, this is consistent with the known function of collagen XII in regulating collagen I architecture in healthy tissues^30,43,44,77,78^ and supports the observed decrease in biomechanical properties of these matrices.

Finally, we sought to determine whether the observed changes in the 3D organotypic matrices as a result of collagen XII depletion would affect cancer cell invasion in the 3D setting. PyMT cancer cells^79^ were seeded onto, and allowed to invade into the CAF-remodelled organotypic matrices following removal of CAFs (see ‘Methods’) (Fig. 6h). While cancer cells readily invaded into shScr-CAF remodelled matrices, this ability was significantly reduced in collagen I matrices remodelled by the shColXII#1 and #3 CAFs (Fig. 6i, j). Similarly, a reduced cancer cell invasion was observed using the 4T1 murine breast cancer cell line (Supplementary Fig. 5F, G), further confirming that collagen XII regulation of collagen I is important in generating pro-invasive microenvironments in primary breast tumours.

**Fig. 7:**
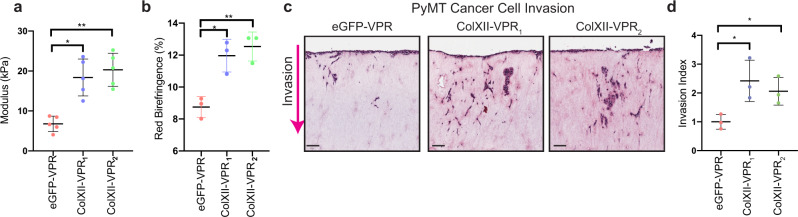
Collagen XII overexpression increases tumour stiffness and cancer cell invasion. **a** Bulk modulus (stiffness) of organotypic matrices at day 12 remodelled by control (eGFP-VPR) or collagen XII overexpressing (ColXII-VPR_1_, ColXII-VPR_2_) CAFs as measured by unconfined compression analysis. Mean ± SD, *n* = 5 biologically independent experiments, *p = 0.032, **p = 0.0075, Kruskal–Wallis test with Dunn’s multiple comparison test. **b** Quantification of the red birefringence signal area for picrosirius-red-stained organotypic matrices generated by control (eGFP-VPR) or collagen XII overexpressing (ColXII-VPR_1_, ColXII-VPR_2_) CAFs. Mean ± SD, *n* = 3 biologically independent experiments, *p = 0.034, **p = 0.0078, one-way ANOVA with a Dunnett’s multiple comparisons test. **c** Representative histological images (H&E) (from *n* = 3 biologically independent experiments) of cancer cell invasion into organotypic matrices remodelled by control (eGFP-VPR) or collagen XII overexpressing (ColXII-VPR_1_, ColXII-VPR_2_) CAFs (scale bar = 100 µm). **d** Quantification of cancer cell invasive index into organotypic matrices remodelled by control (eGFP-VPR) or collagen XII overexpressing (ColXII-VPR_1_, ColXII-VPR_2_) CAFs. Mean ± SD, *n* = 3 biologically independent experiments, *p < 0.05, eGFP-VPR vs ColXII-VPR_1_ *p* = 0.032; eGFP-VPR vs ColXII-VPR_2_ *p* = 0.028; One-way ANOVA with a Dunnett’s multiple comparisons test. Source data are provided in the Source data file.

To confirm that collagen XII expression by CAFs alters matrix biomechanics, fibrillar collagen architecture and cancer cell invasion, we employed a CRISPR-activation (dCas9-CRISPR-VPR) approach with guide RNAs (gRNAs) targeted to two different regions upstream of the collagen XII 5’UTR. This resulted in the generation of two CAF lines that overexpressed collagen XII (gRNA_1_: ColXII-VPR_1_; gRNA_2_: ColXII-VPR_2_) compared with CAFs expressing the non-targeting GFP control gRNA (eGFP-VPR). We confirmed that overexpression of collagen XII by CAFs did not affect the rate of collagen remodelling (Supplementary Fig. 5H) but did significantly increase the stiffness of CAF-remodelled organotypic matrices (Fig. 7a) and the proportion of mature, bundled fibrillar collagens (Fig. 7b and Supplementary Fig. 5I–K). Importantly, matrices remodelled by CAFs overexpressing collagen XII significantly increased the invasion of PyMT cancer cells (Fig. 7c, d). These data demonstrate that increasing collagen XII expression in CAFs exerts an opposite effect to collagen XII depletion, and confirm an important functional role for collagen XII in regulating fibrillar collagen architecture and matrix biomechanics in the tumour microenvironment and subsequently breast cancer cell invasion.

### CAF-secreted collagen XII creates a permissive microenvironment that facilitates metastasis

**Fig. 8:**
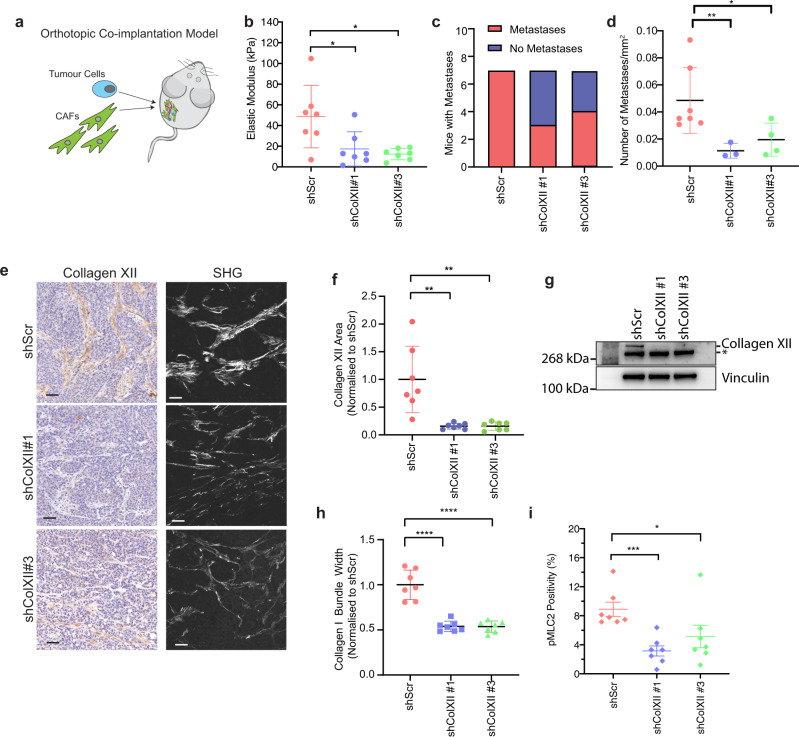
Collagen XII knockdown reduces metastasis in vivo. **a** Schematic representation of orthotopic co-implantation of cancer cells and CAFs (knockdown study: shScr and shColXII; overexpression study eGFP-VPR and ColXII-VPR) injected in a 1:3 ratio respectively. **b** Bulk modulus as determined by unconfined compression analysis of excised primary tumours generated by co-implantation of cancer cells with control (shScr) and collagen XII knockdown (shColXII#1, shColXII#3) CAFs. Mean ± SD, *n* = 7 biologically independent samples, *p < 0.05, shScr vs shColXII#1 *p* = 0.036, shScr vs. shColXII#3 *p* = 0.045; Kruskal–Wallis test with Dunn’s multiple comparisons test. **c** Quantitation of the number of mice with or without observed metastases in H&E sections in the knockdown study (*n* = 7 mice per group). **d** Quantification of the mean number of metastases normalised to the total lung area in mice with metastases present in the knockdown study (*n* = 7 shScr, *n* = 4 shColXII#1 and *n* = 3 shColXII#3 mice with metastases present). *p = 0.0278, **p = 0.0062 two-sided *t* test with Welch’s correction. Data are presented as mean ± SD. **e** Representative images of (left) collagen XII IHC (scale bar = 50 µm) and (right) multi-photon second harmonic generation signal (scale bar = 40 µm) acquired from *n* = 7 primary tumours in the knockdown study. **f** Quantification of collagen XII-positive area in primary tumours from the knockdown study (*n* = 7 mice per group). **p < 0.01 One-way ANOVA with a Dunnett’s multiple comparisons test. Data are presented as mean ± SD. **g** Representative western blot of collagen XII expression in primary tumours at endpoint in the knockdown study of *n* = 2 biologically independent experiments. *Denotes non-specific band. Vinculin is a loading control. **h** Quantification of collagen I fibre bundle width in primary tumours from the knockdown study. Data are presented as mean ± SD, *n* = 7 biologically independent experiments, ****p < 0.0001, one-way ANOVA with Dunnett’s multiple comparisons test. **i** Quantification of pMLC2 stain**i**ng positivity (% of tumour area) in primary tumours. Data are presented as mean ± SD, *n* = 7 mice per group; 3 fields of view per tumour, *p = 0.018, ***p = 0.0006, two-sided Mann-Whitney test. Source data are provided in the Source data file.

Based on our in vitro data, we then investigated the role that collagen XII may be playing in breast cancer development and progression in vivo. To investigate the effects of CAF secreted collagen XII, we orthotopically co-implanted cancer cells with either shScr, shColXII#1 or shColXII #3 CAFs (1:3 ratio cancer cells to CAFs) into the fourth mammary fatpad (Fig. 8a). Following implantation, tumour growth was measured until a maximum endpoint tumour size of 520 mm^3^ (corresponding to 1 cm × 1 cm) was reached. We observed no difference in time to endpoint between scrambled control (shScr) or collagen XII knockdown (shColXII #1 or #3) tumours, with all groups reaching maximum tumour volume within a similar timeframe (Supplementary Fig. 6A, B). Also, tumour weights at endpoint were not significantly different between scrambled control (shScr) and collagen XII knockdown (shColXII #1 or #3) tumours (Supplementary Fig. 6C). Building on our in vitro data suggesting a role for collagen XII in tissue stiffness, we profiled freshly explanted tumours and found a significant decrease in bulk tumour stiffness, measured by unconfined compression analysis (Fig. 8b), in line with our organotypic studies (Fig. 7e).

In light of our in vitro data supporting a role for collagen XII in modulating cancer cell 3D invasion, we assessed metastatic burden within these mice. Macroscopic observation of H&E-stained sections of lung tissue (Supplementary Fig. 6D), one of the major sites of metastasis in breast cancer, revealed that approximately 50% of mice co-implanted with shColXII CAFs did not contain any detectable secondary lung tumours (Fig. 8c), compared to 100% of those co-implanted with shScr CAFs. Further analysis also revealed that in the 50% of mice with collagen XII knockdown CAFs that did exhibit lung metastases, the total number of metastases was significantly reduced (Fig. 8d), although there was no difference in the size of metastases present in mice bearing collagen XII knockdown tumours compared to control (Supplementary Fig. 6E). These data suggest that collagen XII plays a critical role in regulating matrix organisation that facilitates metastatic dissemination of tumour cells.

During primary tumour growth, the recruitment and co-option of host fibroblasts typically occur as has been described in ref. 64. IHC staining for collagen XII demonstrated a sustained significant decrease in collagen XII was still present within the knockdown CAF co-implanted tumours at endpoint compared to scrambled control (Fig. 8e, f and Supplementary Fig. 6F), which was confirmed by western blotting (Fig. 8g). To ensure that this was not due to overall lower numbers of CAFs in the collagen XII knockdown tumours, IHC staining for the CAF marker αSMA^10^ showed no differences in CAF presence within the different tumour groups at endpoint (Supplementary Fig. 6G, H). Thus these data confirm that the lower collagen XII expression in knockdown tumours was not due to a lower overall CAF presence in these tumours, and suggest that recruitment of local fibroblast populations cannot restore collagen XII levels. Furthermore, in line with our in vitro findings, SHG multi-photon imaging of collagen I organisation (Fig. 8e) showed a significant decrease in collagen I bundle thickness (Fig. 8h), correlating with the observed decrease in tumours stiffness.

During tumour progression, fibroblasts within the tumour are known to exhibit increased activation of cytoskeletal contractile machinery and their regulators, including pMLC2, which plays a central role in a positive feedback loop further activating these fibroblasts and amplifying matrix remodelling^10,80^. This matrix remodelling leads to increases in tumour stiffness and the generation of microenvironments that promote tumour cell invasion. Examining the level of MLC2 phosphorylation in primary tumours at endpoint revealed strong pMLC2 staining in the stroma of these tumours. Furthermore, pMLC2 levels were significantly decreased in collagen XII knockdown tumours compared with control tumours (Fig. 8i and Supplementary Fig. 6I), consistent with reduced primary tumour stiffness and subsequently decreased metastatic potential of cancer cells in an environment with low collagen XII expression. These data confirm that collagen XII depletion in CAFs leads to changes in collagen I bundling and tumour stiffness within the developing tumours, that likely leads to an overall lower level of stromal activation and the abrogation of a cancer cell invasion permissive microenvironment.

**Fig. 9:**
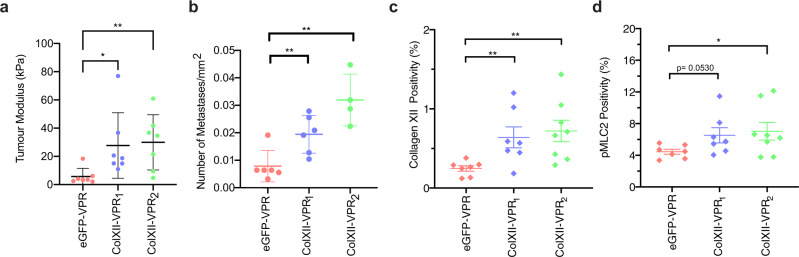
Collagen XII overexpression promotes metastasis in vivo. **a** Bulk modulus as determined by unconfined compression analysis of excised primary tumours generated by co-implantation of cancer cells with control (eGFP-VPR) and collagen XII overexpressing (ColXII-VPR_1_, ColXII-VPR_2_) CAFs. Data are presented as mean ± SD, *n* = 7 eGFP-VPR mice, *n* = 7 ColXII-VPR_1_ and *n* = 8 ColXII-VPR_2_ mice, *p = 0.017, **p = 0.0066, Kruskal–Wallis test with Dunn’s multiple comparisons test. **b** Quantification of mean number of metastases normalised to the total lung area in mice with metastases present in the overexpression study (*n* = 6 eGFP-VPR mice, *n* = 6 ColXII-VPR_1_ and *n* = 4 ColXII-VPR_2_ mice with metastases present). Data are presented as mean ± SD. eGFP-VPR vs. ColXII-VPR_1_ *p* = 0.0087; eGFP-VPR vs. ColXII-VPR_2_ *p* = 0.0048, two-sided Mann–Whitney *U*-test. **c** Quantification of collagen XII-positive area in primary tumours from the overexpression study. *n* = 7 eGFP-VPR mice, *n* = 7 ColXII-VPR_1_ and *n* = 8 ColXII-VPR_2_ mice. **p < 0.01, eGFP-VPR vs. ColXII-VPR_1_ *p* = 0.0098; eGFP-VPR vs ColXII-VPR_2_ *p* = 0.025, two-sided Mann–Whitney *U*-test. One-way ANOVA with a Dunnett’s multiple comparisons test. Data are presented as mean ± SD. **d** Quantification of pMLC2 positive area in primary tumours from the overexpression study. *n* = 7 eGFP-VPR mice, *n* = 7 ColXII-VPR_1_ and *n* = 8 ColXII-VPR_2_ mice. *p = 0.04, two-sided Mann–Whitney *U*-test. Data are presented as mean ± SD. Source data are provided in the Source data file.

Finally, to further validate the functional importance of Collagen XII expression in metastatic dissemination, we orthotopically co-implanted cancer cells with CAFs overexpressing collagen XII (ColXII-VPR_1_, ColXII-VPR_2_) or control CAFs (eGFP-VPR), into the fourth mammary fatpad (1:3 ratio cancer cells to CAFs) similar to the knockdown experiments. Overexpression of collagen XII within the primary tumour was associated with a small increase in primary tumour progression (Supplementary Fig. 7A–C). Consistent with the data from the knockdown study, collagen XII overexpression by CAFs resulted in stiffer tumours (Fig. 9a), concordant with the role of this FACIT collagen in regulating tumour biomechanics. Importantly, overexpression of collagen XII in tumours also led to a higher number of lung metastases compared with control tumours (Fig. 9b), although did not significantly affect the number of mice with lung metastases (Supplementary Fig. 7D, E), nor did it affect the size of metastases present (Supplementary Fig. 7F). Finally, we confirmed that at endpoint, there was a sustained increase in collagen XII expression within the tumour microenvironment as measured by both IHC (Fig. 9c and Supplementary Fig. 7G) and western blotting (Supplementary Fig. 7H, I). Consistent with the knock-down study, there was a trend towards increased pMLC2 staining in tumours overexpressing collagen XII (Fig. 9d and Supplementary Fig 7J), reinforcing collagen XII-induced biomechanical changes in the primary breast tumour microenvironment that support cancer cell metastasis.

Overall, these complementary knockdown and overexpression in vitro and in vivo models support the hypothesis that CAF-secreted collagen XII at the primary site modulates collagen I organisation and tumour biomechanics, thereby creating a metastasis permissive tumour microenvironment.

## Discussion

The development and progression of many solid tumours is accompanied by a desmoplastic response whereby the deposition and remodelling of the tumour matrix lead to significant changes in the biochemistry, biomechanics, architecture, and topography of the tumour microenvironment^41,81,82^. It is well established that the ECM is a salient feature of solid tumours and can play both a pro-tumourigenic^8–13^, as well as anti-tumourigenic role^1,5,14,15^. In particular, the total and relative amounts of each matrix component, as well as the specific 3D supramolecular assembly and organisation are critically important^1,5^. It is clear that increased matrix deposition alone is not always a robust indicator of patient outcome^3^, and given the paradoxical role of the matrix in cancer, recent work has focussed on uncovering the critical elements which regulate matrix assembly and organisation, and the role that they play in solid tumour progression. Many studies have also implicated CAFs^64^ as one of the key architects of matrix deposition and remodelling in solid tumours^83^.

While single timepoint ECM profiling studies have revealed critical insight into how the matrix differs between tumour and non-tumour tissues^31,33,35^, matrix remodelling is a highly dynamic process. Insight into how the matrix changes throughout tumour progression will reveal the individual and collective contribution of key components at critical clinically important stages of tumour progression, such as metastatic dissemination. Here, using matrisome-enriched temporal proteomic profiling, we interrogated the changing tumour matrisome during breast cancer progression in a spontaneous autochthonous immunocompetent model of breast cancer. This identified 4 key matrisomal clusters with different temporal profiles that likely play important and different roles during progression. For example, early upregulation of cluster 2 proteins such as SPP1 may represent a specific matrix remodelling programme associated with initial fibroblast trans-differentiation into CAFs that accelerates tumour progression during the early stages of transformation^50^. Conversely, cluster 1 proteins such as fibulin 5 have also been implicated in promoting cancer cell invasion in other cancer types^84^ and therefore may represent a temporal profile that supports metastatic dissemination.

In particular, we pinpoint the cluster 1 protein collagen type XII as a critical component that is upregulated in tumours compared to healthy tissue, and which is also increasingly deposited over time as tumours progress from hyperplastic lesions to adenoma and through to metastatic adenocarcinoma^85^. Through cross-comparison of our proteomic profiling with single-cell transcriptomic analysis of tumours from matched animal models and patient tumours, we revealed the source of collagen XII as CAFs. We show that collagen XII, a FACIT collagen, is predominantly secreted by CAFs and is important in regulating the spatial organisation of collagen I within the primary tumour microenvironment. Collagen XII is the largest member of the FACIT collagens^30^ and is known to bind to type I collagen fibrils to regulate their organisation and contribute to tissue biomechanics in the cornea, skeletal muscle and tendon^30,43,44,86^. Emerging evidence is now revealing dysregulation of this ECM component in several cancer types. Recently, collagen XII expression has been correlated with high mammographic density in women, a known risk factor for poor outcome in breast cancer^20^. Collagen XII has also previously been associated with poor clinical outcomes in gastric cancer^55^; however, to date, a functional role for collagen XII in breast cancer metastasis had not been reported.

Analysis of both human and murine single-cell transcriptomic data identified CAFs as a major source of collagen XII in the tumour microenvironment, concordant with previous reports of high expression of collagen XII in colorectal carcinoma myofibroblasts in vitro^47^. Importantly, our data indicate that collagen XII expression levels in the primary tumour are not simply a marker of CAF presence within the tumour, but that this CAF-secreted collagen XII plays a functional role in creating an invasion-permissive 3D microenvironment that supports metastatic dissemination of breast cancer cells. Stable manipulation (knockdown or overexpression) of collagen XII expression in CAFs in our co-implantation in vivo orthotopic model significantly altered collagen I organisation and biomechanics of the tumour tissue. This subsequently leads to a significant alteration of dissemination to the lung, with increases in collagen XII accompanying increased metastasis.

Since collagen XII levels significantly affected metastasis number but not time to endpoint in the knockdown study, our data suggest that elevated collagen XII levels in the primary tumour facilitate cancer cells in disseminating to the secondary tissues such as the lung. This increase in metastatic potential may arise from either more cancer cells disseminating from the primary tumour and/or an increased survival advantage that facilitates arrival at the lung. Our in vitro data suggests that collagen XII levels at the primary tumour site modulate the invasiveness of cancer cells, lending support to the former mechanism of metastasis potentiation. Importantly, this suggests that a future intervention targeting collagen XII-cancer cell crosstalk may disrupt the pro-invasive effects of a collagen XII-rich tumour microenvironment. However, since an almost complete depletion of collagen XII did not abrogate metastasis in general, this reinforces the notion that collagen XII is likely one of many elements within the tumour microenvironment affecting dissemination.

Importantly, our data also indicate that in the primary tumour microenvironment, collagen XII regulates collagen I organisation. The organisation of collagen I fibrils, and the associated changes in tissue biomechanics, have been widely implicated in promoting cancer cell invasion and metastasis^2,8,18^, largely through mechanotransduction mechanisms. Importantly, increased stiffness also acts on normal fibroblasts to drive them towards a myofibroblast phenotype that accelerates further tumour-promoting matrix remodelling, a phenotype supported by our pMLC2 data^10,87^. Emerging roles for collagen I architecture and tumour biomechanics in immunosurveillance^88,89^ suggests that the effects of collagen XII on these parameters may have broader implications for immunological constraints on tumour progression. Overall, collagen XII expression has the potential to reinforce matrix remodelling leading to the generation of an environment that facilitates metastatic dissemination.

In analysing expression levels of collagen XII in patient cohorts we found that high expression of collagen XII is significantly associated with poor progression-free and overall survival in breast cancer patients. Importantly, collagen XII was found to be a significant predictor of progression-free survival in early-stage (stage I–II) patients, a population in which overt metastatic dissemination is not detected at the time of diagnosis. Within this population, the prediction of recurrent disease remains a considerable clinical challenge. Perhaps surprisingly, collagen XII expression was the strongest predictor of progression-free survival in this group compared to other known clinicodemographic indicators of risk. Supported by our in vitro and in vivo data, this suggests that in breast cancer, collagen XII may be playing a key role in early dissemination to secondary sites. With further development and validation in independent cohorts, and importantly in a large prospective study, collagen XII expression in the primary tumour stroma may find utility as a biomarker of early dissemination and therefore high risk of recurrence in early-stage breast cancer patients.

Finally, our data also show that matrisomal profiling is a powerful method to reveal dynamic changes in the matrix and understanding these changes may present new underexplored stromal targets that play important roles at critical stages of tumour progression. In recent years, there has been a marked increase in interest in the role of the matrix in defining the properties of the tumour microenvironment, and our findings support the emerging hypothesis that future therapeutic approaches to ‘normalise’ or re-engineer the tumour stroma may offer the potential for significant translational impact in improving patient survival.

## Methods

### Study design

All animal research was conducted according to protocols approved by the St Vincent’s Precinct and Garvan Institute Animal Ethics Committee (protocol # ARA19_08). All research involving biobanked human TMA biospecimens and data was covered by the Royal Prince Alfred Hospital Human Ethics Review Committee Approval (X14-0241). Informed consent had been provided for the study and compensation was not provided. The number of mice used in each experiment is detailed in corresponding figure legends. Wild-type (wt) or polyoma middle-T antigen (PyMT) mammary tissues were harvested at early (8–10 weeks), mid (11–13 weeks) or late (14–16 weeks) timepoints. In the tumour setting, this corresponds to hyperplasia, adenoma, followed by carcinoma^90^. Tissue biomechanical properties were quantified using unconfined compression analysis on freshly collected samples. Tumour and normal tissue were then utilised in either histological or proteomic mass spectrometry workflows. LC-MS/MS was performed on 5 independent biological replicates (with the exception of mid-stage tumour which consisted of 4 biological replicates) in a single proteomic run. CDMs and organotypic contraction and invasion assays were performed in three independent biological repeats with three technical replicates. Picrosirius red, IHC, SHG, GLCM, orientation and fibre bundle width analyses were performed on three regions of interest in CDMs and organotypic matrices and five regions of interest in animal tissues.

### Animal studies and ethics

Female FVB/n mice with or without the polyoma middle-T antigen (PyMT) transgene under the mouse mammary tumour virus (MMTV) promoter were obtained in house^66^ in conventional animal facilities in line with the Australian code of practice for the care and use of animals for scientific purposes, including standard ambient temperature, humidity and dark/light cycles. Genotyping of genetically engineered mouse models was performed by Garvan Molecular Genetics (GMG) facility (Sydney, Australia). For co-implantation studies, female FVB/n mice aged 10–12 weeks were used as detailed below in “Orthotopic in vivo study”. For the knockdown study *n* = 7 mice were used per group. For the overexpression study *n* = 8 mice were used per group; however, one mouse in each of the eGFP-VPR and ColXII-VPR_1_ groups did not develop tumours so was excluded from further analysis. All animal work was carried out in accordance with protocols approved by the St Vincent’s Precinct and Garvan Institute Animal Ethics Committee (protocol # ARA19_08).

### Cell lines and cell culture

wt mammary fibroblasts (NFs) derived from wt FVB/n mice (NFs), or cancer associated fibroblasts from transgenic PyMT FVB/n mice (CAFs) were a kind gift from Fernando Calvo^10^. Fibroblast lines were maintained in Dulbecco’s modified Eagle media (DMEM) supplemented with 10% FBS, 1% ITS (insulin–transferrin–selenium) and 1% penicillin/streptomycin. PyMT 20065 cancer cells were derived with the support of Dr Karen Blyth at the CRUK Beatson Institute in Glasgow through the SEARCHbreast initiative (https://searchbreast.org/), a resource to facilitate sharing of archived material derived from in vivo breast cancer models. PyMT 20065 cancer cells were maintained in DMEM supplemented with 10% FBS, 1% penicillin/streptomycin, 5 µg/mL insulin, 10 ng/mL epidermal growth factor and 10 ng/mL Cholera Toxin A as previously described^79^. All cells were kept at 37 °C in 20% O_2_ and 5% CO_2_. Cells were routinely tested and confirmed negative for mycoplasma.

### Proteomics

#### Proteomic sample preparation

To enrich matrix proteins, tissue was incubated for 16 h with shaking at room temperature in 0.5% sodium deoxycholate (SDC). Samples were centrifuged at 1000 × *g* for 2 min and the supernatant was discarded. Pellets containing decellularized proteins were washed briefly with 0.5% SDC, centrifuged at 1000 × *g* for 2 min and the supernatant discarded. Proteins were resuspended in 1% sodium dodecyl sulfate (SDS) in 100 mM Tris, pH 8.5 and solubilised with 2 × 20 s tip-probe sonication. Protein was quantified using a BCA and normalised to 20 µg/100 µL of 1% SDS in 100 mM Tris, pH 8.5. The samples were reduced and alkylated in a final concentration of 10 mM Tris(2-carboxyethyl)phosphine and 40 mM 2-Chloroacetamide, respectively for 5 min at 45 °C. Peptides were prepared using a modified single-pot, solid-phase-enhanced sample preparation as previously described^91^. Briefly, samples were diluted to 50% ethanol and incubated with 1:1 mixture of hydrophilic:hydrophobic Seramag Speedbead carboxyl magnetic beads (GE Life Sciences) for 8 min at room temperature. The supernatant was removed, and the beads were washed three times with 80% ethanol. The beads were then resuspended in 10% trifluoroethanol in 100 mM Tris-HCl, pH 7.5 and digested with 0.4 µg of sequencing-grade trypsin (Sigma) and 0.4 µg of sequencing grade LysC (Wako, Japan) overnight with shaking at 37 °C. Trypsin digestion was halted by acidification to 1% trifluoroacetic acid (TFA) and purified by styrenedivinylbenzene-reverse phase sulfonate (SDB-RPS) microcolumns. The columns were washed with 99% isopropanol containing 1% TFA followed by 5% acetonitrile containing 0.2% TFA and peptides eluted with 80% acetonitrile containing 1% ammonium hydroxide and dried by vacuum centrifugation. Peptides were resuspended in 2% acetonitrile containing 0.1% TFA and stored at −20 °C prior to running.

#### Mass spectrometry acquisition and data processing

Prepared peptide samples were analysed using a Dionex nanoUHPLC coupled to a Q-Exactive HF-X in positive polarity mode using XCalibur. Peptides were separated on 20 cm × 100 µm column constructed in-house with an integrated emitter and packed with 1.9 µm C18AQ particles (Dr. Maisch, Germany). One microgram of the peptide was injected and eluted over a linear gradient of 3–35% Buffer B over 60 min at 60 °C with a flow rate of 800 nl/min (Buffer A = 0.1% formic acid; Buffer B = 80% acetonitrile, 0.1% formic acid). The mass spectrometer was operated in data-independent acquisition (DIA) using identical settings and variable-sized isolation windows as previously described^92^. DIA data were analysed in Spectronaut Pulsar X using library-free searching. Peptide quantification was performed at MS2 level using 3–6 fragment ions, which included automated interference fragment ion removal as previously described^92^. MS1 mass tolerance was set to 20 ppm and the MS/MS fragment mass tolerance was set to 0.02 Da. Dynamic mass MS1 and MS2 mass tolerance were enabled and local (non-linear) regression was performed for retention time calibration. A dynamic extracted ion chromatogram window size was performed. The minimum peptide length was set to 7 amino acids with specific trypsin cleavage and search criteria included oxidation of methionine and protein N-terminal acetylation set as variable modifications and carbamidomethylation set as a fixed modification. Data were searched against the mouse UniProt (June 2018; 95,128 entries including isoforms) and filtered to 1% FDR at the peptide and protein level (Q-value cut-off <0.01). Peptide quantification was performed using 3–6 fragment ions, and protein quantification was performed with weighted peptide median values. Perseus software was utilised for statistical analysis. Proteins with <70% presence across all samples were filtered in order to yield robustly identified proteins. For a targeted matrix analysis, proteomics data were annotated for matrisome proteins^35^, and non-matrisome proteins were filtered. Differentially abundant proteins were calculated using *t* tests with multiple hypothesis testing using the Benjamini–Hochberg adjustment. Significant differences were defined with an adjusted *p* and *q*  <  0.05. Heatmaps were generated using log_2_ transformed, median-centred *z*-score of protein abundance values, from proteins present in at least 70% of all samples (*n* = 4 or 5). Volcano plot analyses were generated using log_2_ transformed and median-centred protein abundance values of proteins present in at least 70% of all samples (*n* = 4 or 5). Principal component analysis biplots were generated using log_2_ transformed and median-centred protein abundance values, of proteins in all samples (*n* = 4 or 5).

### Unconfined compression analysis

Biomechanical testing of tissues was performed using the TA Instruments Discovery Hybrid Rheometer-3 (DHR3) with TRIOS Data acquisition software. Fresh tumours and mammary glands were isolated at defined timepoints (early [8–10 weeks], mid [11–13 weeks], or late [14–16 weeks]) and immediately subjected to biomechanical testing. Organotypic matrices were profiled after 12 days of remodelling. Compressive elastic modulus was measured by applying a constant linear pressure of 2 µm/s for animal tissues and 10 µm/s for organotypic matrices. The data were acquired and a stress/strain curve for each tissue was obtained. The compressive elastic modulus was obtained from the slope of the linear viscoelastic region of the stress/strain curve accounting for material surface area.

### Immunoblotting

Samples were lysed in protein extraction buffer (50 mM HEPES, 1% Triton-X-100, 0.5% sodium deoxycholate, 0.1% SDS, 0.5 mM EDTA, 50 mM NaF, and protease cocktail inhibitor (Roche)). Proteins were separated by either 4–12% acrylamide Bis-Tris or 3-8% tris-acetate gel electrophoresis (as detailed in individual figure legends), and were transferred onto a PVDF membrane (Immobilon-P, Millipore), blocked with 5% milk, incubated with primary antibodies (COL12A1 1:10,000 Abcam ab121304; B-tubulin 1:10,000 Cell signalling #2146, Vinculin 1:10,000 Sigma V9131, GAPDH 1:10,000 Cell Signalling #2118, clone 14C10) overnight at 4 °C, and probed with HRP-conjugated secondary antibodies (#NA931, GE Healthcare, 1:5000 diluted in 1% milk). Signal was detected using ECL reagent and imaged (Fusion FX, Vilber). Uncropped blots are included in the Supplementary Information File and Source data file.

### Histopathology and immunohistochemistry

Tissues were fixed in 10% buffered formalin and embedded in paraffin and sections were taken at 4 μm. Sections were deparaffinised in xylene and rehydrated using a series of graded ethanol washes. Staining was performed using the Bond RX Autostainer (Leica). Heat-Induced Epitope Retrieval was performed in an EDTA buffer pH 9 (Leica, AR9640) for 40 min at 93 °C. Slides were quenched in Peroxide Block (Leica, DS9800). Primary antibodies were used as follows: COL12A1 (1:150) Abcam ab121304; aSMA (1:150) ab5694 Abcam; pMLC2 (1:100) Cell Signalling Technologies #3671. Staining used the Leica Bond Polymer Refine Detection Kit (Leica, DS9800) as per the manufacturer’s instructions. Skeletal muscle was used as a positive control tissue for optimisation of COL12A1 staining. Staining was visualised using Diaminobenzidine (DAB). H&E staining and counterstaining were performed on the Leica ST5010 Autostainer XL (Leica). Quantification of the DAB area was performed in ImageJ (v2.3.501). Whole stained tissue sections were imaged using an Aperio slide scanner.

### Picrosirius red staining and quantification

Picrosirius red staining was performed as previously described^73^. The area of total collagen stained by picrosirius red was quantified in ImageJ^93^ software using an in-house script as previously published^73^. Briefly, 4 µm sections of fixed samples were deparaffinised, rehydrated, and stained with 0.1% picrosirius red (Polysciences) for fibrillar collagen according to the manufacturer’s instructions. Imaging was performed on Leica DM 6000 (Power Mosaic) at ×20 magnification for animal tissues and ×40 magnification for organotypic collagen matrices. Quantitative measurements of fibrillar collagen signal (Red) were carried out. For each image, Hue-Saturation-Balance (HSB) thresholding was applied, where 200 ≥ *H* ≤ 240 | 150 ≥ *S* ≤ 255 | 0 ≥ *B* ≤ 255 was used. The relative area (as a % of total tissue area) was then calculated.

### SHG imaging

SHG imaging was performed on formalin-fixed paraffin-embedded unstained 4 µm sections as previously described^73^. Collagen I SHG signal was imaged using a 25 × 0.95 NA water objective on an inverted Leica DMI 6000 SP8 confocal microscope. Excitation was achieved using a Ti:Sapphire femtosecond laser (Coherent Chameleon Ultra II) at 80 MHz and tuned to a wavelength of 920 nm. The intensity was recorded with RLD HyD detectors at 460/20 nm. For each subject, five representative regions of interest of 1024 μm × 1024 μm were imaged over a depth of 25.2 µm with a *z*-step size of 2.52 µm. The intensity of the SHG signal was quantified using ImageJ.

### GLCM analysis

Collagen fibre organisation was assessed using GLCM. GLCM analysis was performed using previously published code^73^ as previously described^72,73^. In-house ImageJ and MATLAB scripts are available via GitHub (https://github.com/TCox-Lab). This method quantifies the correlation of SHG signal throughout the matrix, characterising the texture of a sample. The correlation graphs represent the similarity between pixels, where a higher mean correlation demonstrates more organisation within the collagen network, whilst a low correlation demonstrates a less ordered matrix. GLCM analysis was performed in MATLAB (Mathworks, vR2020), using three representative single-plane SHG images. The average texture parameter for each image was calculated using the looped operation of the plug-in and for 0°, 90°, 180° and 270° directions. Normalised texture parameters were calculated for each image, and the mean correlation along with the SEM was imported and plotted in the GraphPad software.

### Collagen fibre bundle width analysis

To measure the width of collagen I fibre bundles, five representative single-plane SHG images were analysed using the Profile Plot function in ImageJ. Outputs generate a signal intensity vs. pixel position histogram, where peak width corresponds to fibre bundle diameter. Within each image, the average fibre bundle width was calculated by analysing the width of collagen fibres in each section of a 3 × 3 grid applied to each ROI.

### Orientation analysis

To quantify collagen I fibre orientation as previously described^31,72,73,94^, an in-house ImageJ script was used. Briefly, structure tensors are utilised to derive the local orientation and isotropic properties of pixels. Tensors were evaluated for each pixel of an input image by computing the continuous spatial derivatives in the *x* and *y* directions using a cubic B-spline interpolation to obtain the local predominant orientation. A hue-saturation-brightness colour-coded map image output is used to indicate the angle of the oriented collagen I within the image. Orientation distribution peaks were then aligned. Distribution shapes denote the degree of linearisation within the image, where wide and broad shapes suggested little coherency in alignment, and narrow peaks imply aligned structures. The ratio between the peak and baseline of each curve was calculated to give the degree of alignment^95^.

### Analysis of COL12A1 expression in patient datasets

#### ‘The Cancer Genome Atlas’ (TCGA)

All gene expression analysis was performed in R (v3.6.1). To assess the relative expression of *COL12A1* in non-tumour and tumour tissue, RNAseq expression values were downloaded from GDAC firehose. The RNAseq data were filtered for lowly expressed genes before being normalised using EdgeR and Limma^96,97^. TCGA RNAseq from the pan-cancer dataset^60^ (RSEM normalised), corresponding clinicodemographic information was obtained from GDAC firehose and survival information was obtained from^98^. *COL12A1* expression was stratified into tertiles, with the upper tertile defined as ‘high’ *COL12A1* expression. Multivariate analyses of *COL12A1* together with clinical covariates included age, stage, a binary variable indicating whether positive lymph nodes were detected and receptor subtype status (Luminal A: ER or PR positive + Her2 negative; Luminal B: ER or PR positive + Her2 negative; Her2: ER and PR negative + Her2 positive; Triple Negative: ER, PR and Her2 negative) as covariates.

#### CAF contribution to survival in the TCGA cohort

A human CAF signature was obtained as the CAF marker genes reported by Wu et al. (2021)^67^. *COL12A1* was removed from this signature to avoid its confounding effect on examining the relationship between *COL12A1* and the CAF signature. A CAF score was generated for each TCGA sample using Gene Set Variation Analysis^99^ and default parameters. The association of the CAF score with overall and progression-free survival was assessed using cox proportional hazards modelling (coxph function of the survminer package in R). Model comparisons were made using the *anova* function of the survival package.

#### Tumour microarrays

Tumour microarrays (TMAs) were constructed from *n* = 150 patients with triple-negative breast cancer (TNBC) as previously described^61^. The TMAs contained at least three cores taken from different areas of each patient's tumour. TMAs were stained by IHC for collagen XII. First, 4 µm sections were deparaffinized in xylene and rehydrated using a series of graded ethanol washes. Staining was performed using the Bond RX Autostainer (Leica). Heat-Induced Epitope Retrieval was performed in an EDTA buffer pH 9 (Leica, AR9640) for 40 min at 93 °C. Slides were quenched in Peroxide Block (Leica, DS9800). The slides were incubated with primary antibodies (COL12A1 1:200 Abcam ab121304) and the Leica Bond Polymer Refine Detection Kit (Leica, DS9800) was used for detection in combination with Diaminobenzidine (DAB). H&E staining and counterstaining were performed on the Leica ST5010 Autostainer XL (Leica). Stained sections were imaged using an Aperio slide scanner. To assess the association of COL12A1 with survival and clinicodemographic features, blinded scoring of COL12A1 IHC staining in stromal compartments of the tumour tissue was carried out by a registered pathologist (Prof Sandra O’Toole, Department of Tissue Pathology and Diagnostic Oncology, Royal Prince Alfred Hospital and NSW Health Pathology, Sydney, NSW, Australia). The maximum COL12A1 score was calculated across each of the three cores per patient and patients were subsequently stratified into high and low groups by the median. The association of COL12A1 stromal scores with disease-specific and distant recurrence was assessed using univariate cox proportional hazards modelling and Kaplan-Meier analysis (Survminer and Survival packages). Univariate Cox proportional hazards modelling of clinical variables identified that Age and lymphatic invasion were significantly associated with measures of survival and therefore, these parameters were included as terms in a multivariate model with the COL12A1 scores in order to assess the independent prognostic importance of COL12A1 expression.

### Single-cell RNA sequencing

#### PyMT mammary tumour

Single-cell RNAseq data of PyMT tumours were obtained from the Valdes-Mora et al. 2021 dataset (GSE158677)^66^ selecting only the MMTV-PyMT genotype containing 11,490 cells from five different tumours. This subset was analysed using the Seurat (v3.2) package^100^, establishing QC thresholds for cell calling of <5% mitochondrial to nuclear gene content and <8000 molecules/cell to exclude doublets. Downstream analysis was performed according to ref. 100, including linear dimensional reduction (PCA), building a K-nearest neighbour (KNN) graph using 30 principal components “FindNeighbors” and default clustering parameters “FindClusters”. Data visualisation was performed using non-linear dimensional reduction UMAP^101^.

#### Human Breast Cancer Atlas

We examined the expression of *COL12A1* in CAFs identified in human breast cancers from the Wu et al. 2021 study^67^ (GSE176078). In brief, this study analysed 26 human primary breast cancers across the three major clinical subtypes (ER+, TNBC and HER2+) using single-cell RNA Sequencing on the Chromium platform (10× Genomics). Cell clusters were annotated using published gene signatures^63^ and canonical markers (for CAFs these were *PDGFRA*, *PDGFRB*, *COL1A1*, *PDPN* and *FAP*). The expression of *COL12A1* represents log-normalised gene expression values.

### Stable knockdown and overexpression of collagen XII in CAFs

Stable knockdown of collagen XII was achieved through lentiviral infection of short-hairpin constructs. Briefly, HEK293T cells were grown to 80% confluence in a 100 mm dish. Cells were transfected with 8 µg of shColXII or shSCR control lentiviral GFP constructs (OriGene, TL500400), together with 4.5 µg pMDLg/pRRE, 6.4 µg pRSV-Rev and 2.7 µg pMD.G third-generation packaging plasmids. Transfection was carried out in Opti-MEM reduced serum media using Lipofectamine 2000 as per the manufacturer’s instructions. Following overnight incubation, media was replaced with DMEM supplemented with 10% FBS and 1% penicillin/streptomycin. After 24 h incubation, the lentivirus-containing media was filtered with 0.45 µm filter ready for infection. CAFs were grown to 40% confluence in DMEM supplemented with 10% FBS, 1% penicillin/streptomycin and 1% insulin-transferrin-selenium. CAFs were infected with viral supernatant in the presence of 4 µg/mL polybrene. shColXII#1, shColXII#2, shColXII#3, shColXII#4, and shSCR expressing CAFs were purified using the FACs Aria III Cell Sorter and FACS Diva software (v8.0.1, BD Biosciences, USA) based on GFP expression. Briefly, CAFs were resuspended in single cell solution and washed twice with FACs buffer (PBS, 2% FBS). Following sorting, CAFs were then plated and expanded. The expression of sh constructs was routinely monitored by profiling GFP expression.

For the generation of overexpression of collagen XII, CAFs were first transduced with the dCas9-VPR-BFP construct using the lentiviral packaging system described above. BFP-positive cells stably expressing the dCas9-VPR construct were captured by FACs as described above and expanded. Lentiviral vectors carrying sgRNAs were generated by restriction cloning of gRNAs into the pLenti-sgRNA-mCherry construct using gRNAs shown in Table 1. BFP-positive cells were then transduced with the gRNA-lentiviral vector using the same packaging system described above. CAFs that were double positive for BFP and mCherry were then isolated by FAC sorting and expanded.

**Table 1:** gRNAs used to generate Col12a1 overexpressing cell lines

Cell Line	gRNA target sequence
eGFP-VPR	GACCAGGATGGGCACCACCC
ColXII-VPR_1_	CGGTGAAGGAGGTCTACTTT
ColXII-VPR_2_	GTAGACCTCCTTCACCGCCG

Collagen XII expression in stable expressing populations was confirmed by qRT-PCR and immunoblotting. Cells were routinely tested and confirmed negative for mycoplasma.

### Real-time quantitative polymerase chain reaction

RNA was isolated using the Macherey–Nagel Nucleospin RNA plus kit as per the manufacturer's instruction. Reverse transcription of RNA was performed using the Quantitect Reverse Transcription Kit (Qiagen) and cDNA was synthesised from 1 µg of total RNA which was diluted 1:10. Experiments were performed using the Roche Universal Probe Library System on the Roche LightCycler 480® (Roche Life Sciences). *Col12a1* expression was detected with the primers forward CCAGGTCCTCCTGGATATTG and reverse AAATTTGTTAGCCGGAACCTG and UPL ProbeLibrary probe 89 (#14689143001, Roche). Expression of the housekeeping gene *RPL19* was detected with primers forward CTCGTTGCCGGAAAAACA and reverse TCATCCAGGTCACCTTCTCA and the UPL ProbeLibrary Probe 103 (#04692217001, Roche). *Col12a1* mRNA expression was normalised to *RPL19* expression and quantified by comparative CT as described previously^94^.

### Cell-derived matrices (CDMs)

CDMs were adapted from previously published protocols^69^. Briefly, CAFs were plated at 2.0 × 10^4^ cells/well in a 24-well tissue culture plate and were allowed to grow for 24 h, before media was supplemented with 50 µg/mL ascorbic acid, changed every 48 h, for 6 days.

### Organotypic assays

Organotypic assays were performed as previously published^73^ following the protocol of ref. 10 and are briefly described below.

#### Contraction assay

Rat-tail collagen I was extracted with 0.5 M acetic acid to a final concentration of 3.0 mg/mL. In all, 3 × 10^5^ CAFs (shSCR, shColXII#1 or shColXII#3) were embedded in rat tail collagen I at a final concentration of 2.0 mg/mL. After polymerisation, the collagen I organotypic plugs were incubated for 12 days in DMEM supplemented with 10% FBS, 1% penicillin/streptomycin and 1% insulin–transferrin–selenium, renewing media on day 6. CAF-remodelled organotypic matrices were then either subjected to biomechanical testing or used in cancer cell invasion assays. Prior to use in invasion assays, pharmacological removal of CAFs was achieved with 400 µg/mL hygromycin for 48 h followed by 3 × 30 min washes in 37 °C phosphate-buffered saline, followed by 1 × 30 min equilibration in DMEM with 10% FBS and 1% penicillin/streptomycin to generate a cell-free matrix that had been remodelled by CAFs.

#### Invasion assay

Following remodelling, 1 × 10^5^ cancer cells were seeded on top of the organotypic matrix in DMEM supplemented with 10% FBS, 1% penicillin/streptomycin, 5 µg/mL insulin, 10 ng/mL epidermal growth factor and 10 ng/mL Cholera Toxin A, and were allowed to settle for 48 h. The organotypic matrix was then transferred to a metal grid establishing an air–liquid interface and cancer cells were allowed to invade for 12 days, with the renewal of DMEM every 72 h. Organotypic matrices were then fixed in 10% formalin and processed for histological analyses. The invasive index was measured in three representative regions per organotypic matrix using the formula below (Eq. 1). Cancer cells were considered to have invaded if they were present at a distance of >50 µm from the upper surface.

1 $${{{{{{\rm{Invasive}}}}}}}\;{{{{{{\rm{index}}}}}}}=\frac{{{{{{{\rm{number}}}}}}}\;{{{{{{\rm{of}}}}}}}\,{{{{{{\rm{invading}}}}}}}\;{{{{{{\rm{cells}}}}}}}}{{{{{{{\rm{number}}}}}}}\;{{{{{{\rm{of}}}}}}}\;{{{{{{\rm{invading}}}}}}}\;{{{{{{\rm{cells}}}}}}}+{{{{{{\rm{number}}}}}}}\;{{{{{{\rm{of}}}}}}}\;{{{{{{\rm{non}}}}}}}{{\mbox{-}}}{{{{{{\rm{invading}}}}}}}\;{{{{{{\rm{cells}}}}}}}}$$

### Orthotopic syngeneic in vivo study

shScr, shColXII#1 or shColXII#3 CAFs and cancer cells were resuspended as single cells and counted on an automated cell counter to obtain 1 × 10^6^ cancer cells and 3 × 10^6^ CAFs (1:3 ratio). Cells were mixed in 50 µL PBS and were kept on ice before being injected into the fourth mammary fatpad of 10-week-old female FVB/n mice. Tumour growth was monitored three times weekly using callipers and tumour volume was calculated using the formula:

2 $${{{{{{\rm{Tumour}}}}}}}\;{{{{{{\rm{volume}}}}}}}=({{{{{{{\rm{Maximum}}}}}}}\;{{{{{{\rm{dimension}}}}}}}}^{2}\times {{{{{{\rm{Minimum}}}}}}}\;{{{{{{\rm{dimension}}}}}}})\times 0.52$$

The maximum tumour volume permitted by our ethics committee was 520 mm^3^ (Eq. 2). The maximal tumour volume was not exceeded. Secondary endpoints were defined as the development of general signs of disease/discomfort (dehydration, prolonged hunching, ruffled coat, fluid built up in the abdomen, abdominal distension, reduced movement/reactivity, obvious lesions or huddling in cage corner), or weight loss ≥10% body weight. In this study, all mice were culled for the primary endpoint. Fresh tumour samples were subjected to biomechanical testing immediately post-collection, before being fixed in 10% formalin and processed for histological analyses. Lungs were inflated with and perfused with Fekete’s solution (580 mL absolute EtOH, 80 mL 37% formalin, 40 mL glacial acetic acid and 300 mL ddH_2_O) for 24 h before being processed and embedded for histological analyses. Quantification of pulmonary metastasis was performed on three-step serial sections taken 250 µm apart for each biological replicate. Metastases were defined as foci of tumour cells present in the lung above 100 µm^2^. To quantify the size of metastases, the area of each focus was measured and normalised to the total cross-sectional area of the lung.

### Statistics and reproducibility

For animal studies, group sizes were determined based on an effect size of 80%, power of 80% and alpha of 5%. An additional mouse in each group was included in the overexpression study to account for up to 10% of orthotopically injected mice that may fail to develop tumours in this model in our experience. Mice that failed to develop tumours were excluded from all analyses. Mice were randomised to treatment groups and were co-housed to minimise selection bias. For in vitro studies, the experimental design precluded any randomisation. Investigators were blinded to treatment groups for the animal experiments for tumour growth measurements. For the imaging analysis of the orthotopic tumours and in vitro organotypic 2D and 3D matrices, all data were analysed identically using automated scripts to minimise selection bias. For all other in vitro studies, investigators were not blinded to allocation during experiments and outcome assessment due to the nature of the experimental design. The number of times experiments were independently repeated is indicated in the figure legends. All attempts to replicate the data were successful.

Unless otherwise stated, non-parametric one-way ANOVA was performed with Dunnett’s multiple comparisons test, and non-parametric two-way ANOVA was performed with Tukey’s multiple comparisons test. Correlations were quantified using Pearson’s correlation test. Kaplan–Meier curves were analysed using a Cox Proportional Hazards Modelling for human data and a log-rank Mantel–Cox test for animal studies. Perseus (v1.6.7.0) was used for multiple sample testing of proteomic data with a false-discovery rate of 0.05. GraphPad Prism v9 was used for all other analyses. Summary data in figures are presented as mean with standard deviation unless otherwise stated. Asterisks denote statistical significance, *p  <  0.05, **p  <  0.01, ***p  <  0.001, ****p  <  0.0001 with precise *p* values indicated in the figure legends.

### Reporting summary

Further information on research design is available in the Nature Research Reporting Summary linked to this article.

## Supplementary information

Supplementary Information

Description of Additional Supplementary Files

Supplementary Data 1

Reporting Summary

## Data Availability

The TCGA RNAseq used in this study is publicly available from the pan-cancer dataset^60^, with additional clinicodemographic information obtained from GDAC firehose and survival information from ref. 98. The publicly available human scRNA-seq data from ref. 67 is publicly available as processed scRNA-seq data for in-browser exploration and download through the Broad Institute Single Cell portal at https://singlecell.broadinstitute.org/single_cell/study/SCP1039, or through the Gene Expression Omnibus under accession number GSE176078. The publicly available mouse scRNA-seq data used in this study are available in the Gene Expression Omnibus database under accession code GSE158677^66^ and https://gallegovaldeslab.shinyapps.io/pymt_shiny/. The matrisome proteomic data generated in this study have been deposited in the MassIVE https://massive.ucsd.edu and ProteomeXchange (www.proteomexchange.org/) databases under accession code PXD032876. The data are publicly available. The processed proteomic data are available in Supplementary Data 1. The remaining data are available within the Article, Supplementary Information or Source data file. Source data are provided with this paper.

## Source data

Source Data

## References

[1] Cox TR (2021). The matrix in cancer. Nat. Rev. Cancer.

[2] Kai F, Drain AP, Weaver VM (2019). The extracellular matrix modulates the metastatic journey. Dev. Cell.

[3] Tian C (2020). Cancer cell-derived matrisome proteins promote metastasis in pancreatic ductal adenocarcinoma. Cancer Res..

[4] Hebert JD (2020). Proteomic profiling of the ECM of xenograft breast cancer metastases in different organs reveals distinct metastatic niches. Cancer Res..

[5] Cox TR, Erler JT (2011). Remodeling and homeostasis of the extracellular matrix: implications for fibrotic diseases and cancer. Dis. Model. Mech..

[6] Cox TR, Erler JT (2016). Fibrosis and cancer: partners in crime or opposing forces?. Trends Cancer.

[7] Pereira BA (2019). CAF subpopulations: a new reservoir of stromal targets in pancreatic cancer. Trends Cancer.

[8] Maller O (2021). Tumour-associated macrophages drive stromal cell-dependent collagen crosslinking and stiffening to promote breast cancer aggression. Nat. Mater..

[9] Levental KR (2009). Matrix crosslinking forces tumor progression by enhancing integrin signaling. Cell.

[10] Calvo F (2013). Mechanotransduction and YAP-dependent matrix remodelling is required for the generation and maintenance of cancer-associated fibroblasts. Nat. Cell Biol..

[11] Peng DH (2020). Collagen promotes anti-PD-1/PD-L1 resistance in cancer through LAIR1-dependent CD8+ T cell exhaustion. Nat. Commun..

[12] Conklin MW (2011). Aligned collagen is a prognostic signature for survival in human breast carcinoma. Am. J. Pathol..

[13] Provenzano PP (2006). Collagen reorganization at the tumor-stromal interface facilitates local invasion. BMC Med.

[14] Bhattacharjee, S. et al. Tumor restriction by type I collagen opposes tumor-promoting effects of cancer-associated fibroblasts. *J. Clin. Invest*. **131**, e146987 (2021). 10.1172/JCI146987 PMC8159701 33905375

[15] Chen Y (2021). Type I collagen deletion in αSMA+ myofibroblasts augments immune suppression and accelerates progression of pancreatic cancer. Cancer Cell.

[16] McConnell JC (2016). Increased peri-ductal collagen micro-organization may contribute to raised mammographic density. Breast Cancer Res..

[17] Huo CW (2015). High mammographic density is associated with an increase in stromal collagen and immune cells within the mammary epithelium. Breast Cancer Res..

[18] Provenzano PP (2008). Collagen density promotes mammary tumor initiation and progression. BMC Med..

[19] Martin LJ, Boyd NF (2008). Mammographic density. Potential mechanisms of breast cancer risk associated with mammographic density: hypotheses based on epidemiological evidence. Breast Cancer Res..

[20] Northey JJ (2020). Stiff stroma increases breast cancer risk by inducing the oncogene ZNF217. J. Clin. Investig..

[21] Boyd NF (2002). Heritability of mammographic density, a risk factor for breast cancer. N. Engl. J. Med..

[22] Boyd NF (2007). Mammographic density and the risk and detection of breast cancer. N. Engl. J. Med..

[23] Sherratt MJ, McConnell JC, Streuli CH (2016). Raised mammographic density: causative mechanisms and biological consequences. Breast Cancer Res..

[24] Conklin MW (2018). Collagen alignment as a predictor of recurrence after ductal carcinoma in situ. Cancer Epidemiol. Biomark. Prev..

[25] Danielson KG (1997). Targeted disruption of decorin leads to abnormal collagen fibril morphology and skin fragility. J. Cell Biol..

[26] Minamitani T (2004). Modulation of collagen fibrillogenesis by tenascin-X and type VI collagen. Exp. Cell Res..

[27] Sottile J, Hocking DC (2002). Fibronectin polymerization regulates the composition and stability of extracellular matrix fibrils and cell-matrix adhesions. Mol. Biol. Cell.

[28] Mouw JK, Ou G, Weaver VM (2014). Extracellular matrix assembly: a multiscale deconstruction. Nat. Rev. Mol. Cell Biol..

[29] Shaw LM, Olsen BR (1991). FACIT collagens: diverse molecular bridges in extracellular matrices. Trends Biochem. Sci..

[30] Chiquet M, Birk DE, Bönnemann CG, Koch M (2014). Collagen XII: protecting bone and muscle integrity by organizing collagen fibrils. Int. J. Biochem. Cell Biol..

[31] Mayorca-Guiliani AE (2017). ISDoT: in situ decellularization of tissues for high-resolution imaging and proteomic analysis of native extracellular matrix. Nat. Med..

[32] Ragelle H (2017). Comprehensive proteomic characterization of stem cell-derived extracellular matrices. Biomaterials.

[33] Gocheva V (2017). Quantitative proteomics identify Tenascin-C as a promoter of lung cancer progression and contributor to a signature prognostic of patient survival. Proc. Natl Acad. Sci. USA.

[34] Naba A (2017). Characterization of the extracellular matrix of normal and diseased tissues using proteomics. J. Proteome Res..

[35] Naba A (2012). The matrisome: in silico definition and in vivo characterization by proteomics of normal and tumor extracellular matrices. Mol. Cell. Proteomics.

[36] Rhim AD (2014). Stromal elements act to restrain, rather than support, pancreatic ductal adenocarcinoma. Cancer Cell.

[37] Özdemir BC (2014). Depletion of carcinoma-associated fibroblasts and fibrosis induces immunosuppression and accelerates pancreas cancer with reduced survival. Cancer Cell.

[38] Becker LM (2020). Epigenetic reprogramming of cancer-associated fibroblasts deregulates glucose metabolism and facilitates progression of breast cancer. Cell Rep..

[39] Joyce MH (2018). Phenotypic basis for matrix stiffness-dependent chemoresistance of breast cancer cells to doxorubicin. Front. Oncol..

[40] Drain, A. P. et al. Matrix compliance permits NF-κB activation to drive therapy resistance in breast cancer. *J. Exp. Med*. **218**, e20191360 (2021). 10.1084/jem.20191360 PMC8025243 33822843

[41] Murphy KJ (2021). Intravital imaging technology guides FAK-mediated priming in pancreatic cancer precision medicine according to Merlin status. Sci. Adv..

[42] Tian C (2021). Suppression of pancreatic ductal adenocarcinoma growth and metastasis by fibrillar collagens produced selectively by tumor cells. Nat. Commun..

[43] Izu Y (2021). Collagen XII mediated cellular and extracellular mechanisms regulate establishment of tendon structure and function. Matrix Biol..

[44] Young BB, Zhang G, Koch M, Birk DE (2002). The roles of types XII and XIV collagen in fibrillogenesis and matrix assembly in the developing cornea. J. Cell Biochem..

[45] Hicks D (2014). Mutations in the collagen XII gene define a new form of extracellular matrix-related myopathy. Hum. Mol. Genet..

[46] Veit G (2006). Collagen XII interacts with avian tenascin-X through its NC3 domain. J. Biol. Chem..

[47] Karagiannis GS (2012). Proteomic signatures of the desmoplastic invasion front reveal collagen type XII as a marker of myofibroblastic differentiation during colorectal cancer metastasis. Oncotarget.

[48] Wu Y, Xu Y (2020). Integrated bioinformatics analysis of expression and gene regulation network of COL12A1 in colorectal cancer. Cancer Med..

[49] Attalla S, Taifour T, Bui T, Muller W (2021). Insights from transgenic mouse models of PyMT-induced breast cancer: recapitulating human breast cancer progression in vivo. Oncogene.

[50] Sharon Y (2015). Tumor-derived osteopontin reprograms normal mammary fibroblasts to promote inflammation and tumor growth in breast cancer. Cancer Res..

[51] Goldoni S (2008). An antimetastatic role for decorin in breast cancer. Am. J. Pathol..

[52] Reed CC (2005). Decorin prevents metastatic spreading of breast cancer. Oncogene.

[53] Shen M (2019). Tinagl1 suppresses triple-negative breast cancer progression and metastasis by simultaneously inhibiting integrin/FAK and EGFR signaling. Cancer Cell.

[54] Liot S (2020). Loss of Tenascin-X expression during tumor progression: a new pan-cancer marker. Matrix Biol..

[55] Jiang X (2019). COL12A1, a novel potential prognostic factor and therapeutic target in gastric cancer. Mol. Med. Rep..

[56] Lattouf R (2014). Picrosirius red staining: a useful tool to appraise collagen networks in normal and pathological tissues. J. Histochem. Cytochem..

[57] Junqueira LC, Bignolas G, Brentani RR (1979). Picrosirius staining plus polarization microscopy, a specific method for collagen detection in tissue sections. Histochem. J..

[58] Chen X, Nadiarynkh O, Plotnikov S, Campagnola PJ (2012). Second harmonic generation microscopy for quantitative analysis of collagen fibrillar structure. Nat. Protoc..

[59] Williams RM, Zipfel WR, Webb WW (2005). Interpreting second-harmonic generation images of collagen I fibrils. Biophys. J..

[60] Cancer Genome Atlas Network. (2012). Comprehensive molecular portraits of human breast tumours. Nature.

[61] Beckers RK (2016). Programmed death ligand 1 expression in triple-negative breast cancer is associated with tumour-infiltrating lymphocytes and improved outcome. Histopathology.

[62] Cazet AS (2018). Targeting stromal remodeling and cancer stem cell plasticity overcomes chemoresistance in triple negative breast cancer. Nat. Commun..

[63] Wu SZ (2020). Stromal cell diversity associated with immune evasion in human triple-negative breast cancer. EMBO J..

[64] Sahai E (2020). A framework for advancing our understanding of cancer-associated fibroblasts. Nat. Rev. Cancer.

[65] Park D, Sahai E, Rullan A (2020). SnapShot: cancer-associated fibroblasts. Cell.

[66] Valdés-Mora, F. et al. Single-cell transcriptomics reveals involution mimicry during the specification of the basal breast cancer subtype. *Cell Rep.* **35**, 108945 (2021). 10.1016/j.celrep.2021.108945 33852842

[67] Wu, S. Z. et al. An integrated multi-omic cellular atlas of human breast cancers. *Nat. Genet*. **81**, 129 (2021).

[68] Su S (2018). CD10+GPR77+ cancer-associated fibroblasts promote cancer formation and chemoresistance by sustaining cancer stemness. Cell.

[69] Cukierman E, Pankov R, Stevens DR, Yamada KM (2001). Taking cell-matrix adhesions to the third dimension. Science.

[70] Franco-Barraza J, Beacham DA, Amatangelo MD, Cukierman E (2016). Preparation of extracellular matrices produced by cultured and primary fibroblasts. Curr. Protoc. Cell Biol..

[71] Franco-Barraza J, Raghavan KS, Luong T, Cukierman E (2020). Engineering clinically-relevant human fibroblastic cell-derived extracellular matrices. Methods Cell Biol..

[72] Vennin C (2019). CAF hierarchy driven by pancreatic cancer cell p53-status creates a pro-metastatic and chemoresistant environment via perlecan. Nat. Commun..

[73] Vennin, C. et al. Transient tissue priming via ROCK inhibition uncouples pancreatic cancer progression, sensitivity to chemotherapy, and metastasis. *Sci. Transl. Med*. **9**, eaai8504 (2017). 10.1126/scitranslmed.aai8504 PMC5777504 28381539

[74] Chitty JL (2020). The Mini-Organo: a rapid high-throughput 3D coculture organotypic assay for oncology screening and drug development. Cancer Rep..

[75] Cicchi R (2010). Scoring of collagen organization in healthy and diseased human dermis by multiphoton microscopy. J. Biophotonics.

[76] Timpson, P. et al. Organotypic collagen I assay: a malleable platform to assess cell behaviour in a 3-dimensional context. *J. Vis. Exp*. e3089 (2011). 10.3791/3089 PMC3227204 22025017

[77] Sun M (2022). Collagen XII regulates corneal stromal structure by modulating transforming growth factor-β activity. Am. J. Pathol..

[78] Schönborn K (2020). Role of collagen XII in skin homeostasis and repair. Matrix Biol..

[79] Floerchinger A (2021). Optimizing metastatic-cascade-dependent Rac1 targeting in breast cancer: Guidance using optical window intravital FRET imaging. Cell Rep..

[80] Wang S (2021). CCM3 is a gatekeeper in focal adhesions regulating mechanotransduction and YAP/TAZ signalling. Nat. Cell Biol..

[81] Alexander J, Cukierman E (2016). Stromal dynamic reciprocity in cancer: intricacies of fibroblastic-ECM interactions. Curr. Opin. Cell Biol..

[82] Beacham DA, Cukierman E (2005). Stromagenesis: the changing face of fibroblastic microenvironments during tumor progression. Semin. Cancer Biol..

[83] Tian C (2019). Proteomic analyses of ECM during pancreatic ductal adenocarcinoma progression reveal different contributions by tumor and stromal cells. Proc. Natl Acad. Sci. USA.

[84] Yue W (2009). Fibulin-5 suppresses lung cancer invasion by inhibiting matrix metalloproteinase-7 expression. Cancer Res.

[85] Naba A, Clauser KR, Lamar JM, Carr SA, Hynes RO (2014). Extracellular matrix signatures of human mammary carcinoma identify novel metastasis promoters. Elife.

[86] Sun M (2020). Collagen XI regulates the acquisition of collagen fibril structure, organization and functional properties in tendon. Matrix Biol..

[87] Wipff P-J, Rifkin DB, Meister J-J, Hinz B (2007). Myofibroblast contraction activates latent TGF-beta1 from the extracellular matrix. J. Cell Biol..

[88] Sun X (2021). Tumour DDR1 promotes collagen fibre alignment to instigate immune exclusion. Nature.

[89] Nicolas-Boluda, A. et al. Tumor stiffening reversion through collagen crosslinking inhibition improves T cell migration and anti-PD-1 treatment. *Elife* **10**, e58688 (2021). 10.7554/eLife.58688 PMC8203293 34106045

[90] Guy CT, Cardiff RD, Muller WJ (1992). Induction of mammary tumors by expression of polyomavirus middle T oncogene: a transgenic mouse model for metastatic disease. Mol. Cell. Biol..

[91] Hughes CS (2019). Single-pot, solid-phase-enhanced sample preparation for proteomics experiments. Nat. Protoc..

[92] Bruderer R (2017). Optimization of experimental parameters in data-independent mass spectrometry significantly increases depth and reproducibility of results. Mol. Cell. Proteomics.

[93] Schneider CA, Rasband WS, Eliceiri KW (2012). NIH Image to ImageJ: 25 years of image analysis. Nat. Methods.

[94] Conway JRW (2017). Three-dimensional organotypic matrices from alternative collagen sources as pre-clinical models for cell biology. Sci. Rep..

[95] Zeltz, C. et al. α11β1 Integrin is induced in a subset of cancer-associated fibroblasts in desmoplastic tumor stroma and mediates in vitro cell migration. *Cancers* **11**, 765 (2019). 10.3390/cancers11060765 PMC6627481 31159419

[96] McCarthy DJ, Chen Y, Smyth GK (2012). Differential expression analysis of multifactor RNA-Seq experiments with respect to biological variation. Nucleic Acids Res..

[97] Ritchie ME (2015). limma powers differential expression analyses for RNA-sequencing and microarray studies. Nucleic Acids Res..

[98] Liu J (2018). An integrated TCGA pan-cancer clinical data resource to drive high-quality survival outcome analytics. Cell.

[99] Hänzelmann S, Castelo R, Guinney J (2013). GSVA: gene set variation analysis for microarray and RNA-seq data. BMC Bioinformatics.

[100] Butler A, Hoffman P, Smibert P, Papalexi E, Satija R (2018). Integrating single-cell transcriptomic data across different conditions, technologies, and species. Nat. Biotechnol..

[101] McInnes, L., Healy, J. & Melville, J. UMAP: Uniform Manifold Approximation and Projection for Dimension Reduction. Preprint at https://arxiv.org/abs/1802.03426 (2020).

