# Supplementary material for: Temporal profiling of the breast tumour microenvironment reveals collagen XII as a driver of metastasis
Source: Nat Commun. 2022 Aug 6;13:4587. doi: 10.1038/s41467-022-32255-7 (PMC9357007; doi:10.1038/s41467-022-32255-7)
Supplement: Supplementary file 1 — Supplementary Information [file 41467_2022_32255_MOESM1_ESM.pdf]

Temporal Profiling of the Breast Tumour Microenvironment Reveals Collagen XII as a Driver of Metastasis

Papanicolaou M*, Parker AL* *et al.*

Supplementary Information

Supplementary Table 1.
Univariate Cox-Proportional Hazards Model of TCGA expression data. Hazard ratios and P-values are derived from cox regression.

**Clinical Outcome**	**Parameter**	**Low N (%)**	**High N (%)**	**HR (95% Confidence Interval)**	**P-value**
Overall Survival	COL12A1 Expression	721 (66.6%)	359 (33.3%)	1.396 (1.003-1.941)	**0.048**
Progression-Free Survival	COL12A1 Expression	721 (66.6%)	359 (33.3%)	1.514 (1.084-2.113)	**0.015**

Supplementary Table 2.
Multivariate model of COL12A1 and clinical covariate association with progression-free survival in early-stage patients only (Stage I and II) in the TCGA cohort. Hazard ratios and P-values are derived from cox regression.

**Parameter**		**HR [95% CI]**	**p-value**
COL12A1		2.228[0.999-4.965]	0.05
Age		0.998 [0.963-1.033]	0.91
Stage	I	Reference	
	II	1.219 [0.469-3.159]	0.68
Lymph Nodes positive		0.883 [0.354-2.204]	0.79
Receptor Subtype	Luminal A	Reference	
	Luminal B	1.771 [0.617-5.082	0.288
	Her2	4.07E-8 [0-0.99]	0.99
	Triple Negative	2.35 [0.908-6.083]	0.078

Supplementary Table 3.
Multivariate model of COL12A1 and clinical covariate association with overall survival across all tumour stages in the TCGA cohort. Hazard ratios and P-values are derived from cox regression.

**Parameter**		**HR [95% CI]**	**p-value**
COL12A1		0.979 [0.468-2.047]	0.956
Age		1.045 [1.017-1.073]	**0.00109**
Stage	I	Reference	
	II	1.272 [0.34-4.749]	0.720
	III	3.106 [0.647-14.91]	0.156
	IV	18.92 [3.12-114.688]	**0.00138**

Lymph Nodes positive		1.713 [0.609-4.8176]	0.307
Receptor Subtype	Luminal A	Reference	
	Luminal B	1.857 [0.719-4.798]	0.2
	Her2	2.22 [0.629-7.83]	0.214
	Triple Negative	3.439 [1.469-8.046]	**0.0044**

Supplementary Table 4.
Multivariate model of COL12A1 and clinical covariate association with progression-free survival across all tumour stages in the TCGA cohort. Hazard ratios and P-values are derived from cox regression.

**Parameter**		**HR [95% CI]**	**p-value**
COL12A1		1.608 [0.861-3.006]	0.136
Age		1.013 [0.99-1.038]	0.24
Stage	I	Reference	
	II	1.15 [0.444-2.988]	0.77
	III	2.991 [0.881-10.158]	0.078
	IV	9.214 [1.966-43.178]	**0.0048**
Lymph Nodes positive		1.319 [0.4799-2.703]	0.768
Receptor Subtype	Luminal A	Reference	
	Luminal B	1.145[0.465-2.822]	0.768
	Her2	0.404 [0.054-3.039]	0.378
	Triple Negative	3.69 [1.867-7.293]	**0.00017**

Supplementary Table 5.
Univariate Cox-Proportional Hazards Model of Disease-Specific Survival of TMA Data. Hazard ratios and P-values are derived from cox regression.

**Parameter**	**HR (95% Confidence Interval)**	**P-value**
Collagen XII Stromal Intensity	1.541 (1.051-2.258)	**0.026**
Age	1.026 (1.006-1.053)	0.050
Tumour Size	1.024 (1.006-1.042)	**0.010**
Lymphatic Invasion	2.524 (1.225-5.201)	**0.012**
Lymph Node Invasion	1.779 (0.833-3.802)	0.137
Vascular Invasion	1.723 (0.828-3.584)	0.146

Supplementary Table 6.

Univariate Cox-Proportional Hazards Model of Distant Recurrence of TMA Data. Hazard ratios and P-values are derived from cox regression.

Parameter	HR (95% Confidence Interval)	P-value
Collagen XII Stromal Intensity	1.516 (1.036-2.219)	**0.032**
Age	1.023 (0.997-1.050)	0.080
Tumour Size	1.018 (0.990-1.037)	0.059
Lymphatic Invasion	1.921 (0.939-3.932)	0.074
Lymph Node Invasion	1.627 (0.774-3.422)	0.199
Vascular Invasion	1.471 (0.701-3.082)	0.307

Supplementary Table 7.
Multivariate Analysis* of Collagen XII staining in TMA data. Hazard ratios and P-values are derived from cox regression.

Outcome	Parameter	HR (95% Confidence Interval)	P-value
Disease-Specific Survival	Collagen XII Stromal Intensity	1.449 (0.974-2.157)	0.067
	Age	1.025 (0.976-1.052)	0.072
	Lymphatic Invasion	2.018 (0.929-4.380)	0.075
	Tumour Size	1.021 (0.999-1.042)	0.050
Distant Recurrence	Collagen XII Stromal Intensity	1.540 (1.03-2.298)	**0.034**
	Age	1.020 (0.994-1.047)	0.14
	Lymphatic Invasion	1.609 (0.748-3.460)	0.22
	Tumour Size	1.020 (0.996-1.04)	0.12

* adjusted for age, tumour size, lymphatic invasion

Supplementary Table 8.
Univariate Analysis of COL12A1 and CAF score with overall- and progression-free survival in the TCGA cohort. Hazard ratios and P-values are derived from cox regression. Bold indicates significant p-value.

Outcome	Parameter	HR [95% CI]	P-value
Overall Survival	COL12A1	1.396 [1.003-1.941]	**0.048**
	CAF score	1.430[0.989-2.06681]	0.0572
Progression-Free Survival	COL12A1	1.514 (1.084-2.113)	**0.015**
	CAF score	1.344 [0.901-2.003]	0.147

Supplementary Table 9.
Comparison of progression-free survival models for COL12A1 and the CAF score in the TCGA cohort.

Comparison	Sequential Variables in the Models	Log likelihood ratio test p-value
1	CAF score	0.147
	CAF score + COL12A1	0.098
2	COL12A1	**0.015**
	COL12A1 + CAF score	0.651

Supplementary Figures and Figure Legends

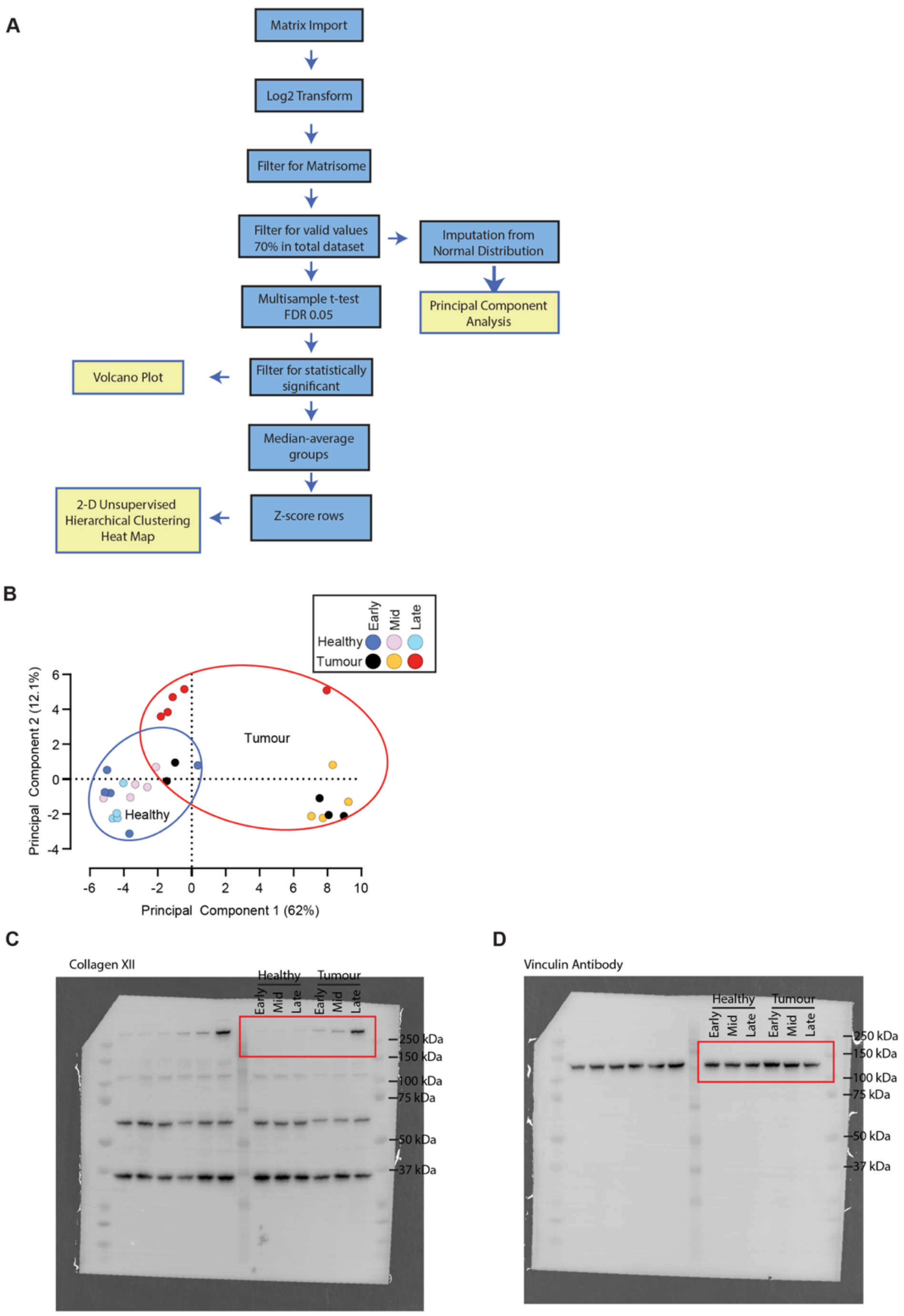

Supplementary Figure 1: Proteomic profiling of decellularized breast tumours reveal dynamic changes in the matrisome. (**A**) Workflow for the analysis of the label-free quantitative liquid chromatography tandem proteomic mass spectrometry (LC-MS/MS) data. (**B**) Principal Component Analysis (PCA) plot of tumour and matched healthy fatpad matrisome data collected at early, mid and late stages of tumour progression. (**C**) Uncropped western blot of collagen XII expression in tumour and matched healthy fatpad tissues corresponding to Figure 2C. Red box indicates cropped region. (**D**) Uncropped western blot of vimentin expression in tumour and matched healthy fatpad tissues corresponding to Figure 2C. Red box indicates cropped region. Source data are provided in the Source Data file.

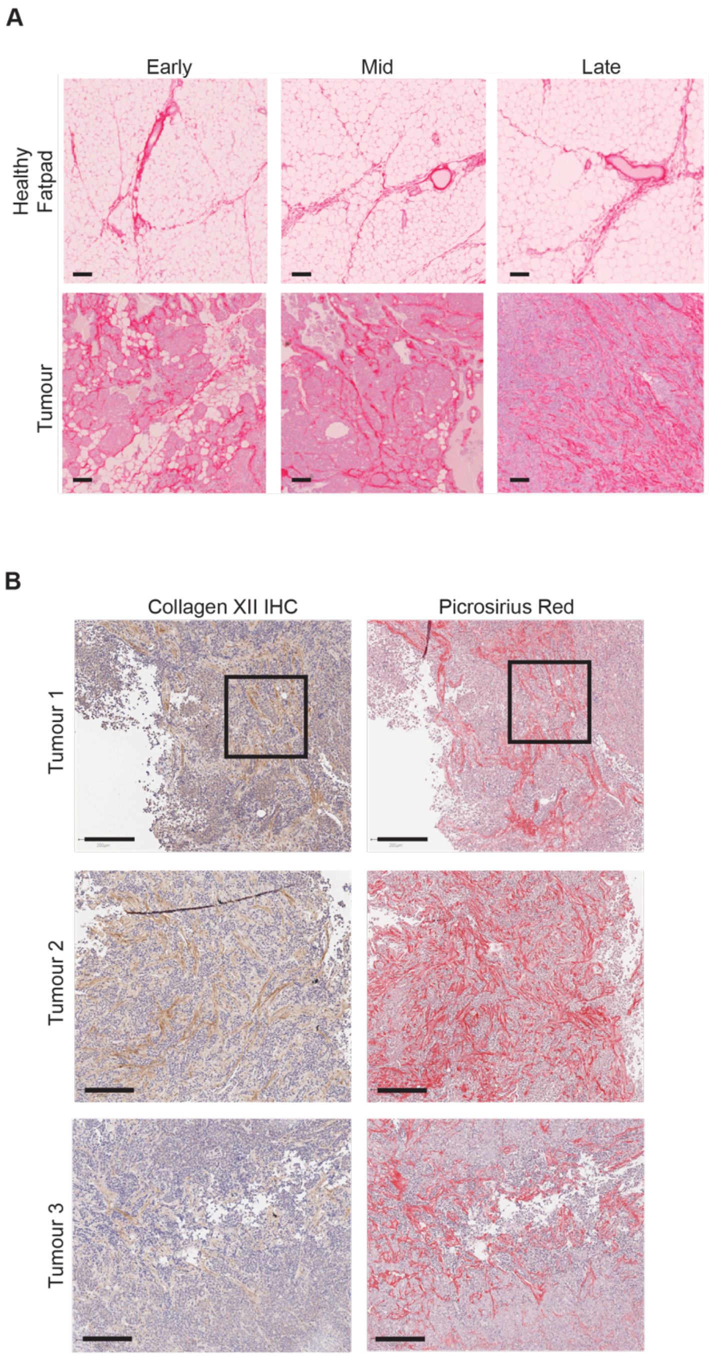

Supplementary Figure 2: Collagen XII associates with fibrillar collagens. (**A**) Representative brightfield images of n=4 picrosirius red-stained tumour and matched healthy fatpad tissues corresponding to Figure 3A and 3B (scale bar = 50 µm). (**B**) Representative collagen XII immunohistochemistry (left panel) and picrosirius red stained tumours corresponding to Figure 3A and 3B (scale bar = 200 µm). n=4. Box indicates field of view shown in Figure 3B. Source data are provided in the Source Data file.

A

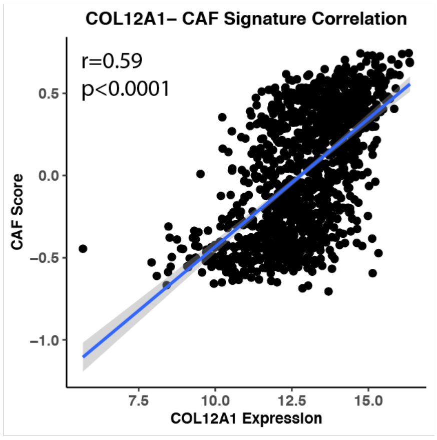

Supplementary Figure 3: Collagen XII expression correlates with the CAF score. (**A**) Correlation of COL12A1 expression with CAFscore in the TCGA dataset. Pearson's correlation test, r=0.59, p<0.0001, n=1,198 patients. Source data are provided in the Source Data file.

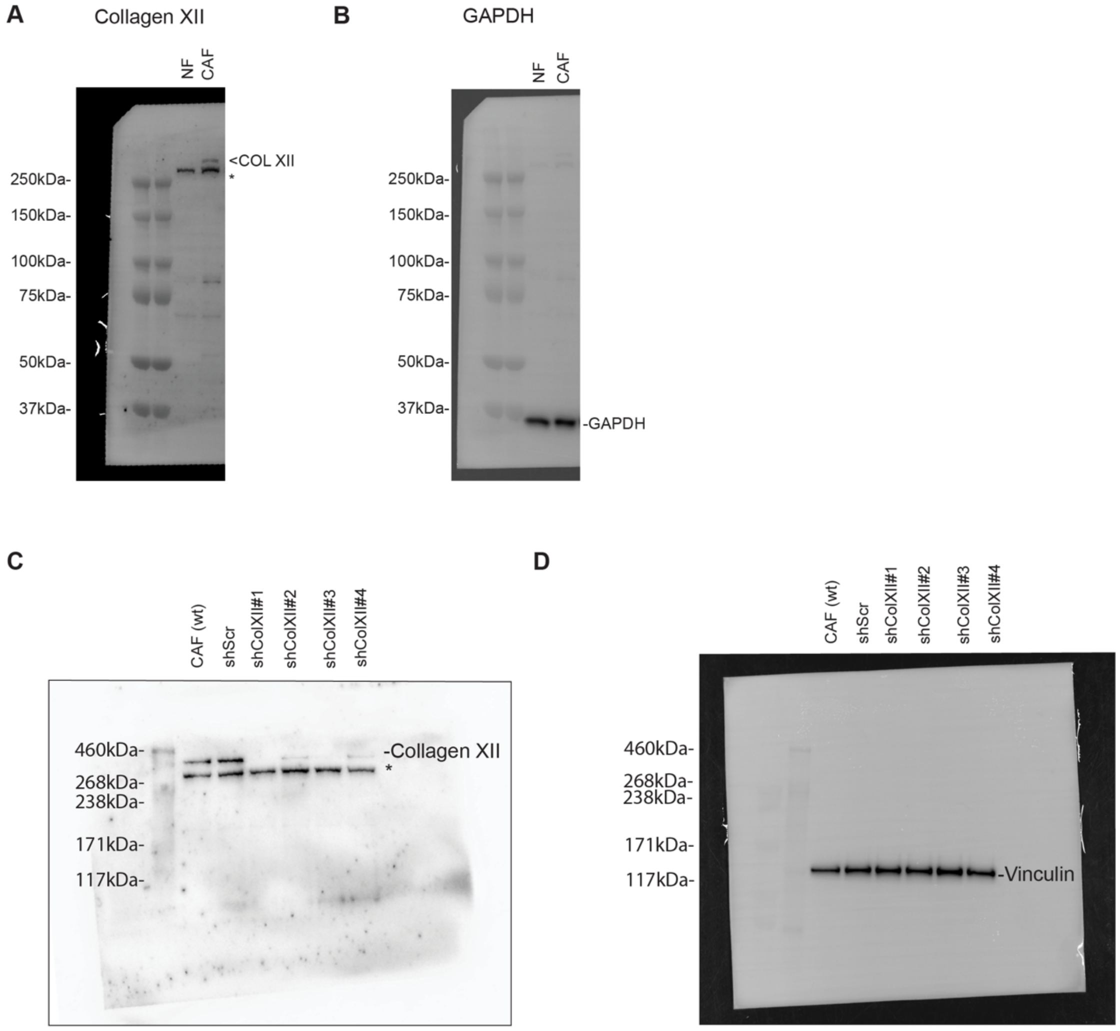

Supplementary Figure 4: Collagen XII is upregulated in cancer-associated fibroblasts. (**A**) Uncropped western blot of collagen XII expression in normal fibroblasts (NF) and PyMT CAFs corresponding to Figure 5G. (**B**) Uncropped western blot of GAPDH expression (loading control) in normal fibroblasts (NF) and PyMT CAFs corresponding to Figure 5G. (**C**) Uncropped western blot of collagen XII expression in normal parental (CAF), control (shScr) and collagen XII knockdown (ColXIIsh#1-4) CAF cell lines corresponding to Figure 5I. * denotes non-specific band. (**D**) Uncropped western blot of vinculin expression (loading control) in normal parental (CAF), control (shScr) and collagen XII knockdown (ColXIIsh#1-4) CAF cell lines corresponding to Figure 5I. * denotes non-specific band. Source data are provided in the Source Data file.

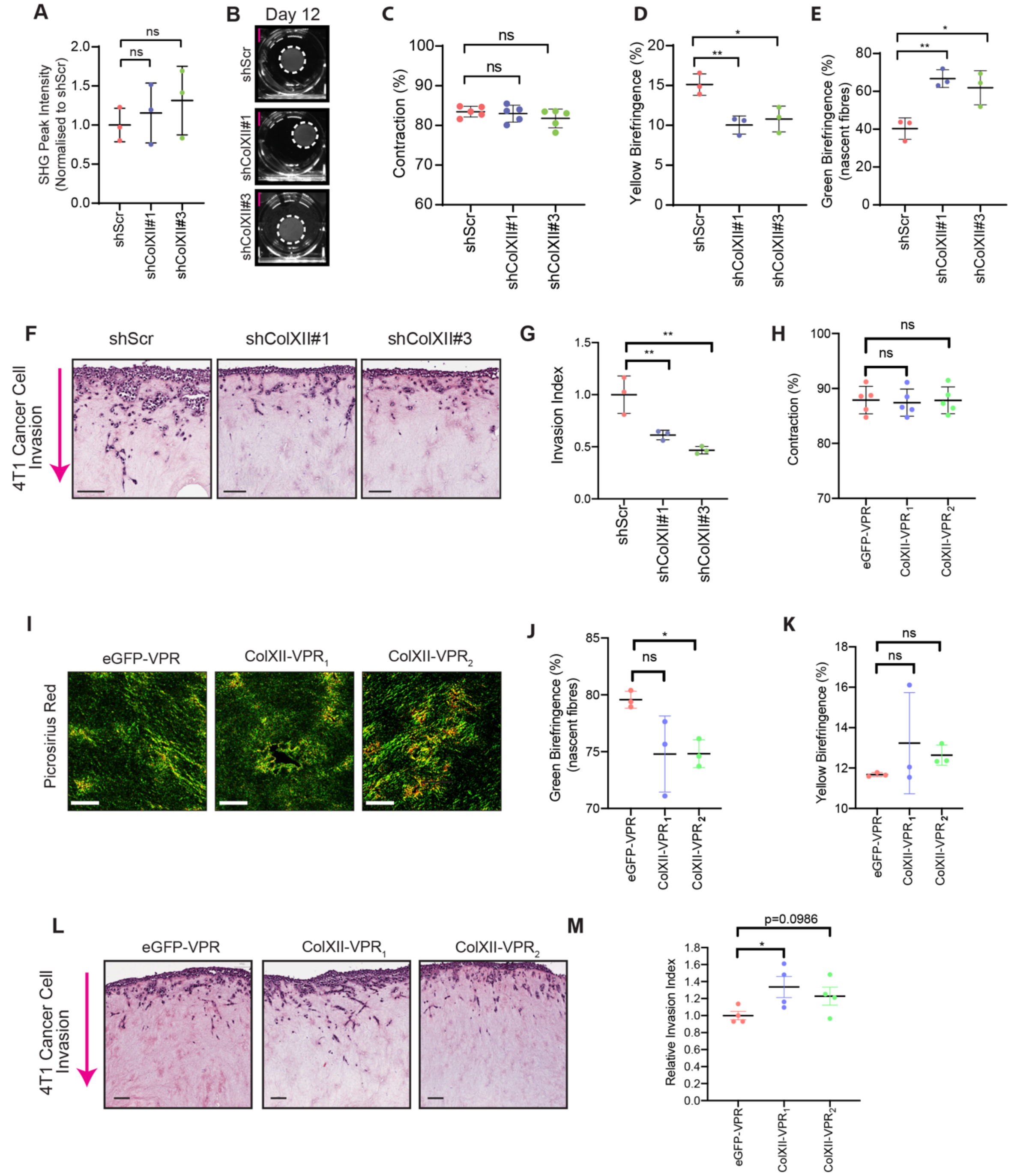

Supplementary Figure 5: Collagen XII modulates fibrillar collagen architecture and promotes cancer cell invasion. (**A**) Quantification of peak SHG multi-photon signal in matrices derived from control (shScr) and collagen XII knockdown (shColXII#1, shColXII#3) CAFs. n=3 biologically independent experiments; ns = not statistically significant. One-way ANOVA with a Dunnett's multiple comparisons test. Data are presented as mean ± SD. Representative images (**B**) and quantitation (**C**) of organotypic matrices remodelled by control (shScr) and collagen XII knockdown (shColXII#1, shColXII#3) CAFs at Day 12. n=3 biologically independent experiments; ns = not statistically significant. One-way ANOVA with a Dunnett's multiple comparisons test. Data are presented as mean ± SD. Quantification of the yellow (**D**) and green (**E**) birefringence signal area for picrosirius-red-stained organotypic matrices

remodelled by control (shScr) or collagen XII knockdown (shColXII#1, shColXII#3) CAFs corresponding to Figure 6G. Mean ± SD, n = 3 biologically independent experiments, Yellow: *p=0.015, **p=0.0073; Green: *p=0.014, **p=0.0053; One-way ANOVA with a Dunnett's multiple comparisons test. (**F**) Representative histological images (H&E) (n=3) of 4T1 mammary cancer cell invasion into organotypic matrices remodelled by control (shScr) or collagen XII knockdown (shColXII#1, shColXII#3) CAFs (scale bar = 100 µm). (**G**) Quantification of 4T1 mammary cancer cell invasive index into collagen XII knockdown organotypic matrices shown in F. Mean ± SD, n = 3 biologically independent experiments, **p<0.01, shScr vs shColXII#1 p=0.0085; shScr vs shColXII#3 p=0.0017; One-way ANOVA with a Dunnett's multiple comparisons test. (**H**) Quantitation of organotypic matrix contraction remodelled by control (eGFP-VPR) or collagen XII overexpressing (ColXII-VPR_1, ColXII-VPR_2) CAFs at Day 12. n=5 biologically independent experiments; ns = not statistically significant. One-way ANOVA with a Dunnett's multiple comparisons test. Data are presented as mean ± SD. (**I**) Representative images of birefringence signal from picrosirius red-stained organotypic matrices remodelled by control (eGFP-VPR) or collagen XII overexpressing (ColXII-VPR_1, ColXII-VPR_2) CAFs imaged under polarising light (scale bar = 100 µm) corresponding to Figure 7B. Quantification of the green (**J**) and yellow (**K**) birefringence signal area for picrosirius-red-stained organotypic matrices remodelled by control (eGFP-VPR) or collagen XII overexpressing (ColXII-VPR_1, ColXII-VPR_2) CAFs corresponding to Figure 7B. Mean ± SD, n = 3 biologically independent experiments, *p=0.019, ns=not significant, Yellow: eGFP vs ColXII-VPR_1 p=0.58, eGFP-VPR vs ColXII-VPR_2 p=0.13; Green: eGFP vs ColXII-VPR_1 p=0.22; One-way ANOVA with a Dunnett's multiple comparisons test. (**L**) Representative histological images (H&E) (n=3) of 4T1 mammary cancer cell invasion into organotypic matrices remodelled by control (eGFP-VPR) or collagen XII overexpressing (ColXII-VPR_1, ColXII-VPR_2) CAFs (scale bar = 100 µm). (**M**) Quantification of 4T1 mammary cancer cell invasive index into collagen XII overexpressing organotypic matrices shown in L. Mean ± SD, n = 4 biologically independent experiments, *p=0.045, two-sided t-test. Source data are provided in the Source Data file.

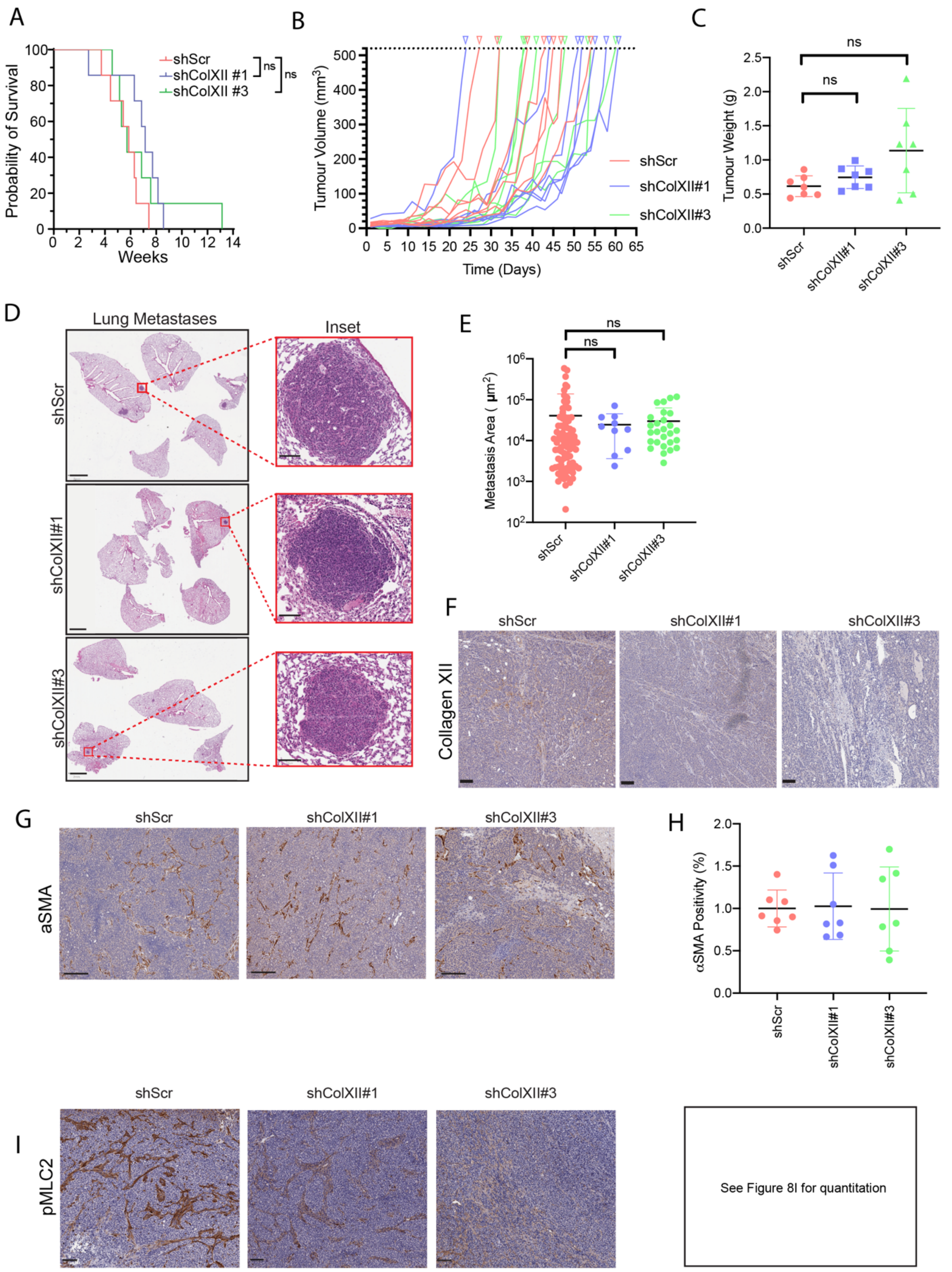

Supplementary Figure 6: Knockdown of collagen XII expression reduces metastasis in PyMT breast tumours. (**A**) Kaplan-Meier analysis of time to endpoint for mice with tumours established by cancer cells co-implanted with control (shScr) or collagen XII knockdown (shColXII#1, shColXII#3) CAFs. N=7 mice per group. Ns = not statistically significant by log-rank test. (**B**) Individual primary tumour volume measurements in the knockdown study. (**C**)

Primary tumour weight at endpoint in tumours generated by cancer cells co-implanted with control (shScr) or collagen XII knockdown (shColXII#1, shColXII#3) CAFs. N=7 mice per group. Data are presented as mean ± SD. Kruskal-Wallis test with Dunn's multiple comparisons test. (**D**) Representative images of (*left*) H&E-stained lungs showing pulmonary metastases and (right) inset zoom of metastatic deposits (3 histological sections from each mouse acquired 250 µm apart) in the knockdown study. (**E**) Quantification of the mean area of metastatic foci in the lung in the knockdown study. NS = not statistically significant, One-way ANOVA with Dunnett's multiple comparisons test. Quantitation of 85 foci identified in n=7 shScr mice that had metastases, 10 foci in n=3 shCOLXII#1 mice that had metastases and 27 foci in n=4 shColXII#3 mice that had metastases. Data are presented as mean ± SD. **F**) Representative images of collagen XII IHC staining (scale bar = 50 µm) of primary tumours established by cancer cells co-implanted with control (shScr) or collagen XII knockdown (shColXII#1, shColXII#3) CAFs. N=7 mice per group. Representative images (**G**) and quantitation (**H**) of alpha-SMA IHC staining (scale bar = 50 µm) of primary tumours established by cancer cells co-implanted with control (shScr) or collagen XII knockdown (shColXII#1, shColXII#3) CAFs. N=7 mice per group. Not statistically significantly different by One-way ANOVA with Dunnett's multiple comparisons test. **I**) Representative images of pMLC2 IHC staining (scale bar = 50 µm) of primary tumours established by cancer cells co-implanted with control (shScr) or collagen XII knockdown (shColXII#1, shColXII#3) CAFs. N=7 mice per group. Corresponding to Figure 8I. Source data are provided in the Source Data file.

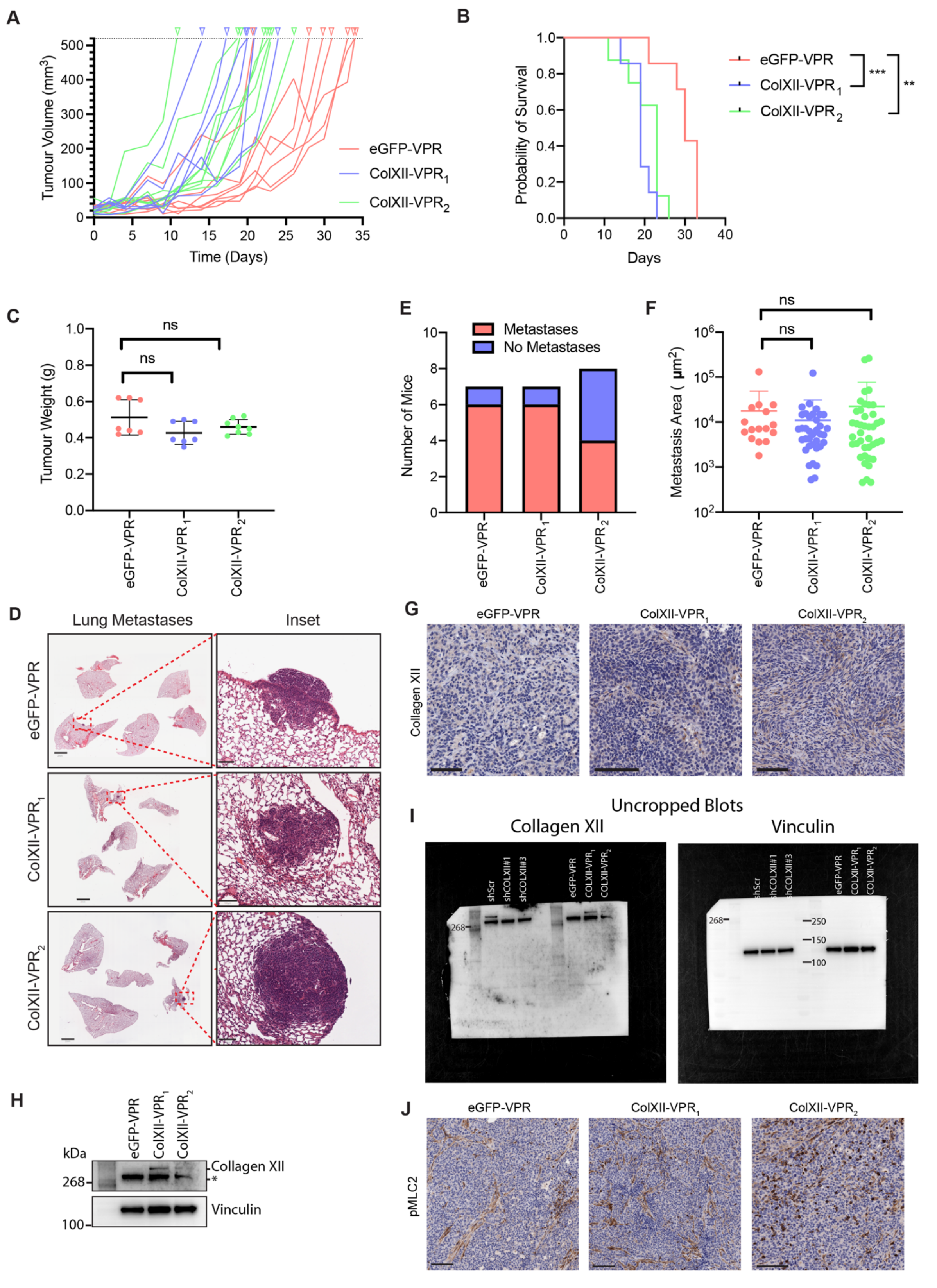

Supplementary Figure 7: Overexpression of collagen XII expression promotes metastasis in PyMT breast tumours. (**A**) Individual primary tumour volume measurements for tumours established by cancer cells co-implanted with control (eGFP-VPR) or collagen XII overexpressing (ColXII-VPR$_1$, ColXII-VPR$_2$) CAFs (overexpression study). (**B**) Kaplan-Meier analysis of time to endpoint in the collagen XII overexpression study. n=7-8 mice per group.

eGFP vs ColXII-VPR$_1$ p=0.0003, eGFP-VPR vs ColXII-VPR$_2$ p=0.001; log-rank test. (**C**) Primary tumour weight at endpoint in tumours generated by cancer cells co-implanted with control (eGFP-VPR) or collagen XII overexpressing (ColXII-VPR$_1$, ColXII-VPR$_2$) CAFs. n=7 eGFP-VPR mice, n=7 ColXII-VPR$_1$ and n=8 ColXII-VPR$_2$ mice. Data are presented as mean ± SD. Kruskal-Wallis test with Dunn's multiple comparisons test. (**D**) Representative images of (*left*) H&E-stained lungs showing pulmonary metastases and (right) inset zoom of metastatic deposits (3 histological sections from each mouse acquired 250 µm apart) in the overexpression study. **E**) Quantitation of the number of mice with or without observed metastases in H&E sections in the overexpression study (n=7 eGFP-VPR mice, n=7 ColXII-VPR$_1$ and n=8 ColXII-VPR$_2$ mice per group). (**F**) Quantification of mean number of metastases normalised to the total lung area in mice with metastases present in the overexpression study (n=6 eGFP-VPR mice, n=6 ColXII-VPR$_1$ and n=4 ColXII-VPR$_2$ mice with metastases present). Kruskal-Wallis test with Dunn's multiple comparisons test. **G**) Representative images of collagen XII IHC staining (scale bar = 100 µm) of primary tumours established by cancer cells co-implanted with control (eGFP-VPR) or collagen XII overexpressing (ColXII-VPR$_1$, ColXII-VPR$_2$) CAFs. n=7 eGFP-VPR mice, n=7 ColXII-VPR$_1$ and n=8 ColXII-VPR$_2$ mice. Data are presented as mean ± SD. (**H**) Representative western blot of collagen XII expression in n=2 primary tumours at endpoint in the overexpression study. * denotes non-specific band. Vinculin is a loading control. (**I**) Uncropped western blot of n=2 collagen XII (left panel) and vinculin (right panel; loading control) expression in primary tumours at endpoint in the knockdown and overexpression studies. * denotes non-specific band. Vinculin is a loading control. Corresponding to Figure 8G and Supplementary Figure 7H. **J**) Representative images of pMLC2 IHC staining (scale bar = 100 µm) of primary tumours established by cancer cells co-implanted with control (eGFP-VPR) or collagen XII overexpressing (ColXII-VPR$_1$, ColXII-VPR$_2$) CAFs. n=7 eGFP-VPR mice, n=7 ColXII-VPR$_1$ and n=8 ColXII-VPR$_2$ mice. Source data are provided in the Source Data file.
