# Supplementary material for: Temporal profiling of the breast tumour microenvironment reveals collagen XII as a driver of metastasis
Source: Nat Commun. 2022 Aug 6;13:4587. doi: 10.1038/s41467-022-32255-7 (PMC9357007; doi:10.1038/s41467-022-32255-7)
Supplement: Supplementary file 2 — Description of Additional Supplementary Files [file 41467_2022_32255_MOESM2_ESM.pdf]

Description of Additional Supplementary Files

File Name: Supplementary Data 1
Description: Proteomic analysis of PyMT mammary tumours throughout progression.
