# Supplementary material for: Temporal profiling of the breast tumour microenvironment reveals collagen XII as a driver of metastasis
Source: Nat Commun. 2022 Aug 6;13:4587. doi: 10.1038/s41467-022-32255-7 (PMC9357007; doi:10.1038/s41467-022-32255-7)
Supplement: Supplementary file 4 — Reporting Summary [file 41467_2022_32255_MOESM4_ESM.pdf]

nature research

Corresponding author(s): Thomas R. Cox
Paul Timpson

Last updated by author(s): Jul 1, 2022

Reporting Summary

Nature Research wishes to improve the reproducibility of the work that we publish. This form provides structure for consistency and transparency in reporting. For further information on Nature Research policies, see our Editorial Policies and the Editorial Policy Checklist.

Statistics

For all statistical analyses, confirm that the following items are present in the figure legend, table legend, main text, or Methods section.

n/a	Confirmed	
☐	☒	The exact sample size (*n*) for each experimental group/condition, given as a discrete number and unit of measurement
☐	☒	A statement on whether measurements were taken from distinct samples or whether the same sample was measured repeatedly
☐	☒	The statistical test(s) used AND whether they are one- or two-sided<br>*Only common tests should be described solely by name; describe more complex techniques in the Methods section.*
☐	☒	A description of all covariates tested
☐	☒	A description of any assumptions or corrections, such as tests of normality and adjustment for multiple comparisons
☐	☒	A full description of the statistical parameters including central tendency (e.g. means) or other basic estimates (e.g. regression coefficient) AND variation (e.g. standard deviation) or associated estimates of uncertainty (e.g. confidence intervals)
☐	☒	For null hypothesis testing, the test statistic (e.g. *F*, *t*, *r*) with confidence intervals, effect sizes, degrees of freedom and *P* value noted<br>*Give P values as exact values whenever suitable.*
☒	☐	For Bayesian analysis, information on the choice of priors and Markov chain Monte Carlo settings
☒	☐	For hierarchical and complex designs, identification of the appropriate level for tests and full reporting of outcomes
☐	☒	Estimates of effect sizes (e.g. Cohen's *d*, Pearson's *r*), indicating how they were calculated

Our web collection on statistics for biologists contains articles on many of the points above.

Software and code

Policy information about availability of computer code

Data collection	Mass Spec data was collected using XCalibur (Thermo)<br>Bulk modulus data was collected using TRIOS Data acquisition software (TA Instruments)
Data analysis	Data analysis was carried out using Microsoft Excel (v16.5) Prism v9, R version 3.6.1, and MATLAB R2020<br>Mass Spec data was analysed in Spectronaut Pulsar X and with Perseus (v1.6.7.0).<br>FACS data was analysed using FACS Diva software (v8.0.1).<br>In house ImageJ(v2.3.501) and MatLab (R2020) scripts for analysis of collagen structure image by SHG and PicRed are available via GitHub (https://github.com/TCox-Lab)

For manuscripts utilizing custom algorithms or software that are central to the research but not yet described in published literature, software must be made available to editors and reviewers. We strongly encourage code deposition in a community repository (e.g. GitHub). See the Nature Research guidelines for submitting code & software for further information.

Data

Policy information about availability of data

All manuscripts must include a data availability statement. This statement should provide the following information, where applicable:
- Accession codes, unique identifiers, or web links for publicly available datasets
- A list of figures that have associated raw data
- A description of any restrictions on data availability

The TCGA RNAseq used in this study are publicly available from the pan-cancer dataset62, with additional clinicodemographic information obtained from GDAC firehose and survival information from reference 98. The publicly-available human scRNAseq data from Wu et al. 2021 [reference 51]:

is publicly available as processed scRNA-seq data for in-browser exploration and download through the Broad Institute Single Cell portal at https://singlecell.broadinstitute.org/single_cell/study/SCP1039. or through the Gene Expression Omnibus under accession number GSE176078 (https://www.ncbi.nlm.nih.gov/geo/query/acc.cgi?acc=GSE176078).The publicly-available mouse scRNAseq data used in this study are available in the Gene Expression Omnibus database under accession code GSE15867750 (https://www.ncbi.nlm.nih.gov/geo/query/acc.cgi) and https://gallegovaldeslab.shinyapps.io/pymt_shiny. The matrisome proteomic data generated in this study have been deposited in the MassIVE https://massive.ucsd.edu and ProteomeXchange (www.proteomexchange.org/) databases under accession code PXD032876 here (http://proteomecentral.proteomexchange.org/cgi/GetDataset?ID=PXD032876). The data is publicly available. The processed proteomic data are available in Supplementary Table 1. The remaining data are available within the Article, Supplementary Information or Source Data file.

Field-specific reporting

Please select the one below that is the best fit for your research. If you are not sure, read the appropriate sections before making your selection.

☒ Life sciences ☐ Behavioural & social sciences ☐ Ecological, evolutionary & environmental sciences

For a reference copy of the document with all sections, see nature.com/documents/nr-reporting-summary-flat.pdf

Life sciences study design

All studies must disclose on these points even when the disclosure is negative.

Sample size	For animal studies, group sizes were determined based on an effect size of 80%, power of 80% and alpha of 5%. An additional mouse in each group was included in the overexpression study to account up to 10% of orthotopically injected mice that may fail to develop tumours in this model in our experience.
Data exclusions	Mice who did not develop tumours were excluded from further analysis.
Replication	Orthogonal validation of findings using complementary approaches were used to validate findings, for example knockdown of Collagen XII was confirmed at both the transcript and the protein level. The number of times experiments were independently repeated is indicated in the figure legends. All attempts to replicate the data were successful.
Randomization	Animals were randomised prior to tumour implant. For non-animal experiments, the experimental design meant that randomisation was not applicable.
Blinding	Animals were monitored by a blinded investigator for tumour growth measurements. Blinding was not possible for in vitro experiments as each independent experiment was carried out by an individual investigator. However, all image analysis was performed using automated scripts to minimise selection bias.

Reporting for specific materials, systems and methods

We require information from authors about some types of materials, experimental systems and methods used in many studies. Here, indicate whether each material, system or method listed is relevant to your study. If you are not sure if a list item applies to your research, read the appropriate section before selecting a response.

Materials & experimental systems

n/a	Involved in the study	
☐	☒	Antibodies
☐	☒	Eukaryotic cell lines
☒	☐	Palaeontology and archaeology
☐	☒	Animals and other organisms
☒	☐	Human research participants
☒	☐	Clinical data
☒	☐	Dual use research of concern

Methods

n/a	Involved in the study	
☒	☐	ChIP-seq
☒	☐	Flow cytometry
☒	☐	MRI-based neuroimaging

Antibodies

Antibodies used	Primary antibodies (COL12A1 1:10,000 Abcam ab121304; Vinculin 1:10,000 Sigma V9131, GAPDH 1:10,000 Cell Signalling #2118, aSMA: 1:150 ab5694 abcam, pMLC2: 1: 100 Cell Signalling Technologies #3671)<br>Secondary antibodies were Amersham ECL Mouse IgG, HRP-linked (1:5000, #NA931, GE Healthcare)
Validation	All antibodies were commercially available from established and verifiable suppliers. No additional validation was undertaken.<br><br>Statements from the manufacturers:<br>Vinculin antibody has been 'independently validated". No other specific validation statements were provided for the other antibodies.

Eukaryotic cell lines

Policy information about cell lines

Cell line source(s)	CAFs and wt mammary fibroblasts (NFs) derived from wt FVB/n mice (NFs), or transgenic PyMT FVB/n mice (CAFs) were a kind gift from Fernando Calvo (Calvo et al. (2013) Nature Cell Biology). The PyMT 20065 cancer cell line was sourced from Professor Karen Blyth CRUK Beatson Institute through the SEARCHBreast initiative (https://searchbreast.org/) and has been previously characterised and published (Floerchinger A et al. Cell Reports (2021)). The 4T1 mammary carcinoma cells were obtained from ATCC (CRL-2539).
Authentication	No authentication undertaken
Mycoplasma contamination	Cells were routinely tested and confirmed negative for mycoplasma.
Commonly misidentified lines (See ICLAC register)	No commonly misidentified cell lines were used in this study.

Animals and other organisms

Policy information about studies involving animals; ARRIVE guidelines recommended for reporting animal research

Laboratory animals	Female FVB/n mice with or without the polyoma middle-T antigen (PyMT) transgene under the mouse mammary tumour virus (MMTV) promoter. Wild type mice used for orthotopic studies were implanted at 10-12 weeks of age and were housed in conventional animal facilities in line with the Australian code of practice for the care and use of animals for scientific purposes, including standard ambient temperature, humidity and dark/light cycles.
Wild animals	No wild animals used
Field-collected samples	No field collected samples used
Ethics oversight	All animal work was carried out in accordance with protocols approved by the St Vincent's Precinct and Garvan Institute Animal Ethics Committee (protocol # ARA19_08).

Note that full information on the approval of the study protocol must also be provided in the manuscript.
